# Measuring Cancer Hallmark Mediation of the TET1 Glioma Survival Effect with Linked Neural-Network Based Mediation Experiments

**DOI:** 10.1038/s41598-020-65369-3

**Published:** 2020-06-01

**Authors:** Thomas Luechtefeld, Nole Lin, Channing Paller, Katherine Kuhns, John J. Laterra, Joseph P. Bressler

**Affiliations:** 1 Insilica LLC, 2736 Quarry Heights Way, Baltimore, MD USA; 2 Johns Hopkins Sidney Kimmel Comprehensive Cancer Center, Baltimore, MD USA; 3 Department of Environmental Health and Engineering, Bloomberg School of Public Health, Johns Hopkins University, Baltimore, MD USA; 4 Kennedy-Krieger Institute, Baltimore, MD USA

**Keywords:** Cancer genomics, Computational biology and bioinformatics, Cancer

## Abstract

This paper examines the effect of TET1 expression on survival in glioma patients using open-access data from the Genomic Data Commons. A neural network-based survival model was built on expression data from a selection of genes most affected by TET1 knockdown with a median cross-validated survival concordance of 82.5%. A synthetic experiment was then conducted that linked two separately trained neural networks: a multitask model estimating cancer hallmark gene expression from TET1 expression, and a survival neural network. This experiment quantified the mediation of the TET1 survival effect through eight cancer hallmarks: apoptosis, cell cycle, cell death, cell motility, DNA repair, immune response, two phosphorylation pathways, and a randomized gene sets. Immune response, DNA repair, and apoptosis displayed greater mediation than the randomized gene set. Cell motility was inversely associated with only 12.5% mediated concordance. We propose the neural network linkage mediation experiment as an approach to collecting evidence of hazard mediation relationships with prognostic capacity useful for designing interventions.

## Introduction

Gliomas represent approximately 75% of primary brain tumors in adults. The median survival of adult patients with gliomas is less than five years, with overall survival of 8–14 months for patients with glioblastoma multiforme (GBM)^1^. The current standard of care includes surgery followed by radiation therapy and treatment with temozolomide. The therapy causes cells to die as a result of extensive DNA damage. Factors that contribute to recurrence are glioma invasiveness, the growth of cell populations resistant to radiation and temozolomide, and the blood brain barrier^2,3^. Classifications of gliomas have been adding molecular features to histology for better diagnosis, especially under circumstances when histological phenotype is unclear. For example, the revised World Health Organization classifications now include 1p/19q co-deletion as a defining feature of oligodendroglial tumors^4^. The use of molecular features will result in more precise prognosis and in the design of treatments tailored to the tumor. Resistance to the alkylating agent temozolomide is associated with promoter methylation of the gene encoding the DNA repair protein O6-methylguanine-DNA methyltransferase^5^.

We recently reported that glioma cell lines deficient with Tet methylcytosine dioxygenase 1 (TET1) exhibited greater genomic instability and were more resistant to ionizing radiation therapy^6^. TET1 belongs to a family of enzymes comprised of three members that catalyzes the conversion of 5-methylcytosine (5-mC) to 5-hydroxymethylcytosine (5-hmC), which is the initial step of DNA demethylation^7^. We found that TET1-deficient cells displayed an attenuated DNA damage response (DDR). TET1-deficient cells fail to undergo apoptosis in response to ionizing radiation and have attenuated DNA repair resulting in high numbers of DNA strand breaks in the survival cell population^6,8^. There has also been evidence indicating the involvement of TET1 in glioma growth in humans. The levels of 5-hydroxymethyl cytosine were strongly depleted in astrocytomas compared to a normal brain^9^. In a study examining the Cancer Genome Atlas, the levels of 5hmC were reported to be associated with poor survival in anaplastic glioma^10^ and GBM^11^.

The hypothesis tested here is that genes involved in the DDR mediate the poor prognosis of glioma patients with low levels of TET1. To test the hypothesis, we modeled mediation relationships between TET1, glioma survival, and eight cancer hallmarks using a linked neural network experiment and data from the Genomic Data Commons. Neural networks demonstrate strong predictive capacity in diverse tasks, such as image classification, speech recognition, and survival models^12–14^. These models are typically trained using observational data and, as a result, sometimes cannot discern correlative relationships from causal ones^15^. The mediation experiment described here is meant to enhance survival neural networks by generating causal hypotheses. To test mediation results, mutation data was analyzed in the Genomic Data Commons (GDC) and cell motility was evaluated in a wound-healing assay. We report that genes involved in the DDR and cell motility mediate the TET1 effect on survival. Additionally, we found a strong association between the effect of TET1 on survival and immune response genes.

## Results

### TET1 saturation in a single variable survival neural network

**Figure 1:**
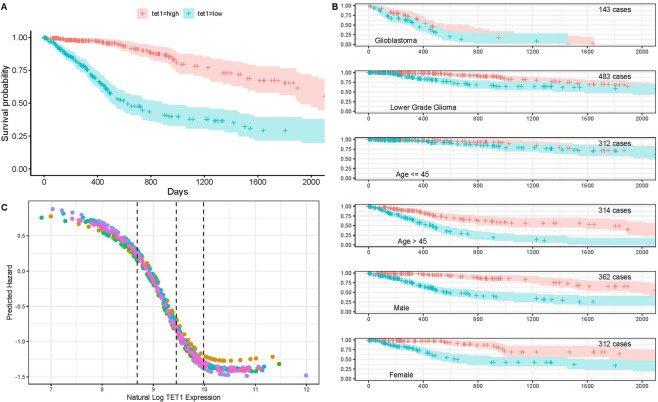
The effect of TET1 expression on glioma patient survival. **(A)** Kaplan-Meier curves for glioma patients with TET1 tumor expression at levels greater than the median (red) and less than the median (blue). **(B)** Kaplan-Meier curves for each for TET1 higher/lower than median by disease type, age and gender. **(C)** Predicted hazard as a function of Log TET1 expression for 10 cross-validated TET1 only models.

The first series of experiments were constructed to determine the relation between survival and TET1 expression. Survival probability curves were developed for patients with high/low TET1 expression (tet_high_/tet_low_) relative to the median expression. TET1 was analyzed directly with GDC RNA-Seq expression data. The 50% survival for tet_low_ is about 650 days, while 50% survival probability for tet_high_ is greater than 2,000 days (Fig. 1A). The relationship between TET1 and patient survival was observed in both GBM and low-grade gliomas (Fig. 1B), and both male and female patients saw similar survival curves. Interestingly, the relationship was much weaker in patients younger than 45 years of age.

A single input variable survival neural network, the TET1 only model, was built from TET1 expression alone to illustrate how neural networks can estimate hazard. The SurvivalNet package was used to build this model with two 10 unit hidden layers and a Cox proportional hazards output layer^16^. The network transforms TET1 expression values into patient hazard. Higher hazard means shorter predicted survival time. Figure 1C shows the hazard functions that are learned by training TET1 only models on 10 non-intersecting partitions of the patient data. In all 10 models, a reduction in hazard is estimated as TET1 expression approached the median value (middle black line). Hazard is highest in the first quartile of expression (left of first dotted line) and lowest (right of last dotted line) in the 4th quartile of expression. No post-processing was performed on the neural network hazard curves in Fig. 1C. The resulting curves show strong consistency despite training on randomized non-overlapping patient partitions.

In cross-validation, the TET1 only model achieved 72.5% average concordance. Each of the training iterations shows a flattening of the hazard curve above a natural log TET1 expression of 10. Flattening hazard above a sufficient level of expression may indicate a saturation effect where TET1 expression gives no additional benefit above a saturation value.

### Confounders affecting the TET1 effect on survival

We investigated some patient and tumor characteristics that might modify the effect that TET1 has on glioma patients. TET1 expression is greater in younger (<45 years) than older patients (Supplemental Fig. 1A) TET1 is differentially expressed with histological type and has greater expression in low-grade tumors than in GBM (Supplemental Fig. 1B). The expression of TET1 is greater in tumors that harbor isocitrate dehydrogenase (IDH) mutations (Supplemental Fig. 1C) in both GBM and low-grade gliomas (Supplemental Fig. 1D). These correlations indicate that the observed TET1 hazard is confounded by age, histology, and IDH1 status.

A multivariate Cox proportional hazards model was constructed on TET1 quartile expression (Q3/4, Q2, and Q1 refer to quartiles, with Q1 the lowest) to evaluate whether TET1 hazard remains significant after controlling for age, IDH1 mutation status, and histology. The lowest quartile of TET1 expression is significantly associated with glioma hazard in a multivariate model of TET1 + age + IDH1 status + Histological type. The reduction in TET1 significance in the multivariate model (Supplemental Fig. 2B) relative to the univariate model (Supplemental Fig. 2A) indicates partial confounding of the TET1 survival effect and these factors.

Forest plots were conducted (Supplemental Fig. 3) to track the significance and confidence intervals of TET1 hazard intervals from the six Kaplan-Meier curve plots in Fig. 1. Expression quartiles are derived from the full patient set and not for each set of patients. The TET1 hazard ratio is significant at the 0.001 level for Q1 in all patient categories (lower-grade gliomas, patients under/over 45, male/female patients) except in GBM patients, where a trend was observed.

To further investigate the effect of confounders, patient-set-specific forest plots were computed using multivariate Cox proportional hazards models. In patients with lower-grade gliomas (LGG) and patients over 45, the TET1 hazard ratio remains significant. In all other patient sets, the TET1 hazard ratio remains positive for Q1, the lowest quartile of expression (Supplemental Fig. 4). These results are weakened by the relatively small patient counts in each category.

### TET1 differentially expressed genes preserve the TET1 survival effect

To strengthen the TET1 only model, we looked for genes causally related to TET1. The use of many related genes in the same model allows for creation of more complex functions of survival. Genes were selected from an earlier study^6,8^ in which gene expression was compared between a human glioma cell line that was made TET1-deficient and a control cell line (Supplemental Table 1). We selected 20 genes with the largest differential expression. These genes were further filtered to 11 by removing those genes with incomplete RNA-seq expression data in the GDC expression files (Table 1). Notably, the selection did not rely on any prior knowledge of prognostic relevance.

**Table 1:** Genes selected for the TET1 affected genes model and their fold change in TET1 knockout experiment.

HGNC	Fold	Concordance	Gene Description
*RAI14*	5.9	69.6	Retinoic acid induced 14
*HGSNAT*	5.5	64.1	Heparan-alpha-glucosaminide N-acetyltransferase
*PLAG24A*	5.5	60.4	Phospholipase A2 group IVA
*PARD3B*	5.5	71.4	Par-3 family cell polarity regulator beta
*ALS2CR11*	5.5	50.4	Amyotrophic lateral sclerosis 2 chromosome region candidate 11
*DALRD3*	−3.8	67.5	DALR anticodon binding domain containing 3
*MUC2*	−3.7	58.4	Mucin 2, oligomeric mucus/gel-forming
*ERCC5*	−3.6	73.6	ERCC excision repair 5, endonuclease
*GUSBP1*	−3.6	50.3	Glucuronidase, beta pseudogene 1
*RALYL*	−3.4	55.9	RALY RNA binding protein-like
*SIX6*	−3.5	57.3	SIX homeobox 6

**Fold** gives the average fold change of these genes in a TET1 knockdown experiment.

**Concordance** gives the concordance of a univariate Cox proportional hazards model built on each respective gene.

A TET1 affected genes model was built with SurvivalNet to evaluate the association between the affected gene expression and survival. TET1 and each of the 11 TET1 affected genes were used. While the purpose of this work is to generate mediation associations of the TET1 affected genes model, it is informative to test the concordance of this model and compare it to the more traditional Cox proportional hazards model. The median concordance increases from ~72.5% for the TET1 only model to 82.5% in the TET1 affected genes model. A Cox proportional hazards model evaluated on the same 10 train and test folds achieves 79.6% mean concordance and is not significantly worse.

The TET1-only survival effect is preserved in the model built from affected genes. When the TET1 only model correctly ranked a pair of patients, the TET1 affected genes model also correctly ranked those patients 90% of the time. When the TET1 model incorrectly ranked a pair of patients, the affected genes model also incorrectly ranked those patients 45% of the time. The shared accuracy indicates that the majority of the observed TET1 survival effect is captured in the TET1 affected genes model. The shared inaccuracy indicates that the TET1 affected genes model is somewhat specific to the TET1 survival effect.

### Cancer hallmarks model survival

**Figure 2:**
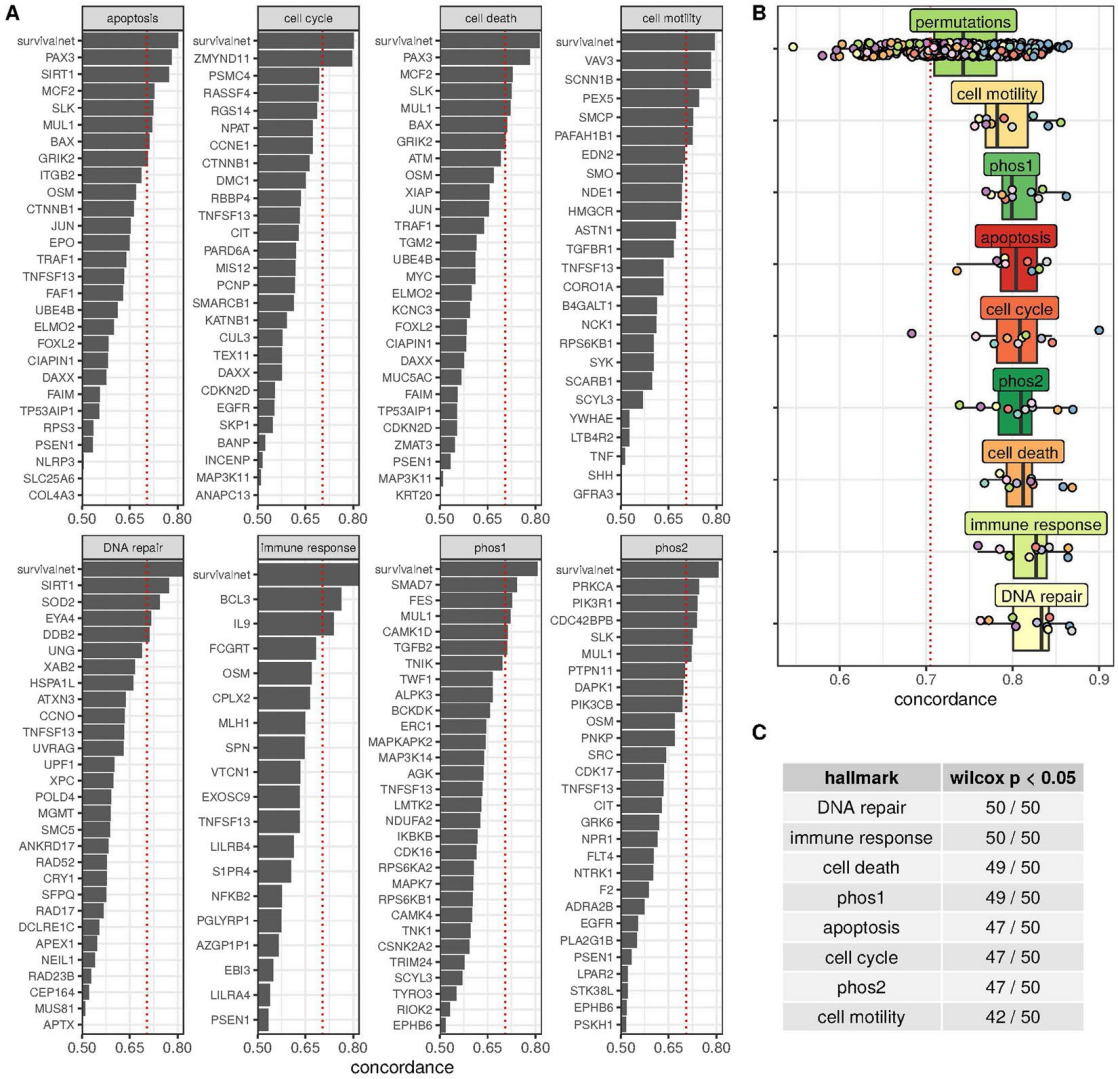
Cox proportional hazard models for cancer hallmark genes. (**A**) Gene membership of each evaluated hallmark and univariate concordance evaluated by Cox proportional hazards model of gene expression. Hallmark SurvivalNet concordance results included under “survivalnet”. **(B)** Five-fold cross-validated concordance values for survival networks built on genes in the respective hallmark (many randomized evaluations for permutations). **(C)** The number of 50 random gene sets the given hallmark model performs better than at a P = 0.05 level in a one tailed Wilcoxon test.

The next series of experiments investigated whether the TET1 effect on survival was mediated by sets of genes termed cancer hallmarks. The term “hallmark” was coined to indicate gene families involved in tumor growth, metastasis, and immune response evasion^17^. Hallmark gene sets were selected from a prior publication focusing on identification of prognostic hallmark-based gene signatures^18^. Eight hallmarks (shown in Fig. 2A) were used to model survival: (1) apoptosis, (2) cell cycle, (3) cell death, (4) cell motility, (5) DNA repair, (6) immune response, (7) phosphorylation pathway 1, and (8) phosphorylation pathway 2. Each hallmark gene set was reduced to 20–30 genes by removing those without complete expression data in some GDC patients. Univariate Cox proportional hazards models were built for every gene in each hallmark for gliomas. Figure 2A gives the concordance of each single gene model. These metrics help indicate which genes have the greatest contribution to the concordances of survival networks that were built for each hallmark. The concordances for each gene in each hallmark were within the range of 0.5 to 0.75. Survival networks built for each hallmark had greater cross-validated median concordances than any univariate gene concordance.

A ninth “permutation” pathway was added consisting of random selections of 10 genes to test whether the concordance was due to chance. Every hallmark model had cross-validated median concordance greater than (1) the median concordance of 50 models built from random gene sets (“permutations” hallmark), and (2) a concordance derived from a univariate Cox proportional hazards model of International Classification of Diseases for Oncology ICD_O_3 histological type (0.7) (Fig. 2B). The DNA repair and immune response models achieved the highest median concordances

Models were compared to the permutation experiments via repeated one-sided Wilcoxon signed ranked tests (Fig. 2C). This statistical test was shown to be the preferred statistical measure for comparing cross-validated model evaluations^19^. The immune response and DNA repair hallmarks performed better in cross-validation at the p = 0.05 level against every repetition of the permutation test, strongly indicating that these gene sets are better than random selections for modeling survival. Every other hallmark except cell motility performed almost as well or better than the false discovery rate against all 50 random gene set experiments.

The association between each hallmark model and the TET1 affected model were evaluated in a mediation experiment. Here, mediation refers to the degree by which the effect of TET1 on survival may be carried out through an intermediary signal, such as the expression of hallmark genes. This neural network-based mediation model is framed on similar experiments done with linear models and described in more detail in the methods.

**Figure 3:**
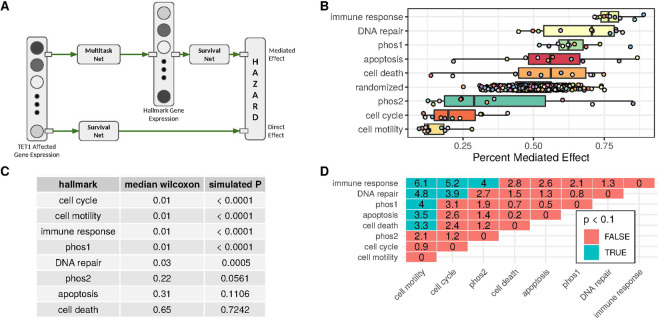
Cancer hallmark gene sets mediate the effects of TET1 on survival. (**A**) Diagram of mediation experiment. The top path is the “Mediated Effect,” wherein a multitask neural network is linked with a survival network to model hazard. The bottom path is the “Direct Effect,” wherein a survival network trains to predict patient hazard from expression of TET1 affected genes. **(B)** The “Percent Mediated Effect” measured in cross-validation for each hallmark and a randomization experiment. **(C)** Median Wilcoxon and simulated p-value for each hallmark’s percent mediated effect compared to randomized hazard percent mediated effects.

The mediated effect measurement examines whether the TET1 effect on survival is mediated by one or more of the cancer hallmarks. The mediation experiments ask the question: “What is the patient hazard associated with the given hallmark if the hallmark genes take on expression values predicted by TET1?” For high concordance to be maintained in the mediation experiment, the TET1 multitask gene expression models must predict hallmark gene expression values that work well with the pre-trained hallmark survival models. Additionally, the pre-trained hallmark survival models must align well with the TET1 direct model. Mediation is determined by inputting predicted expression values from multitask models into the pre-trained hallmark survival networks. The concordance of this composed model is then computed on the set of patient pairs ranked correctly by the TET1 and affected genes’ survival net. This concordance is reported as the percent mediated effect. For a given hallmark, the simulated mediation experiment takes place in two parts: measurement of a hallmark-mediated survival effect, and comparison to the direct survival effect attributable to TET1 and affected genes. In the direct effect, TET1 and its affected genes are collected as an input vector into a survival network that predicts hazard. This is the bottom path in Fig. 3A, and it is the same experiment performed in the section on TET1 survival effect mediation by TET1 affected genes. The top path in Fig. 3A (mediated effect) is made up of two steps. First, a multitask neural network is constructed to predict hallmark gene expression from TET1 affected genes. The multitask neural network is made up of an input layer with TET1 and its affected genes, two fully connected ReLU feed forward hidden layers, and an output for each hallmark gene. Independently, survival networks are constructed for each hallmark and trained directly on hallmark gene expression values. These are the same hallmark survival networks that were described previously.

The models for both the directed and mediated effects generate numeric hazard values for each patient. A metric of mediation, the percent mediated effect mentioned previously, is derived from the percentage of pairs of patients correctly ranked by the direct model that are also correctly ranked by the mediated model.

A percent mediated effect near 1 indicates that the survival effect attributed to the TET1 model is maintained by the given hallmark model, even after transforming the hallmark models inputs via TET1 predicted expression. For a given hallmark, this means that if the input genes take values predicted by TET1 expression, then the existing hallmark model will correctly rank patients who are also correctly ranked by TET1 models – demonstrating that the TET1 survival effect is preserved and that the hallmark is a potential mediator.

If the percent mediated effect is near zero, then patients correctly predicted by the TET1 model are incorrectly predicted in the mediation experiment more often than would be expected by chance. This indicates that the associated hallmark may be an inverse mediator, i.e., the survival effect associated with TET1 is reversed if hallmark genes take on values conditioned on TET1 expression.

Figure 3B displays the percent mediated effect derived in cross-validation for each endpoint. A random hazard function was used as a control and denoted by “randomized”. In the random control, each patient is given a random hazard value sampled from the standard normal distribution. This is done 50 times across 10 folds of cross-validation, so 500 points are visualized in the figure. The DNA repair and immune hallmarks showed percent mediated effects significantly greater than the randomized hazard hallmark. The cell motility hallmark showed a percent mediated effect significantly less than the randomized hazard hallmark. The variance in the DNA repair gene set was large and might be due to the selection of genes examined. The genes in this set cover several different pathways that work independently and in response to different types of damage (see discussion). Two-sided Wilcoxon tests generated 50 p-values from comparison of hallmarks to each random hazard function. The median Wilcoxon p-value (column 2 in Fig. 3C) was calculated for each hallmark. This was then repeated for 10,000 random hazard functions against each of the 50 random hazard functions shown in Fig. 3C to generate a simulated distribution of random hazard function median p-values. The “simulated P” column in Fig. 3C shows the result of this comparison, where cell cycle, cell motility, and immune response outperform all 10,000 random hazard functions. DNA repair outperforms almost all random hazard functions with a median p-value of 0.03.

### DNA repair mediates the TET1 effect on survival

**Figure 4:**
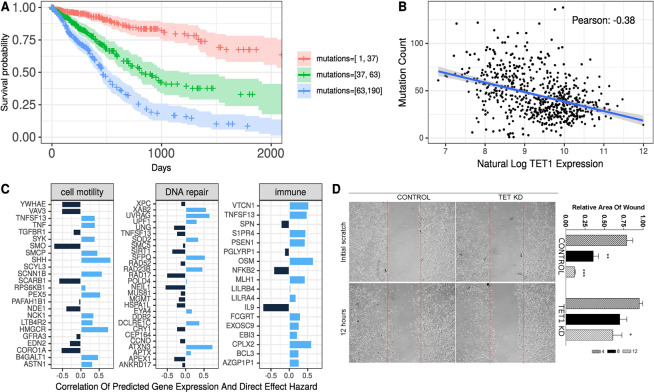
Mutations and cell motility and the TET1 effect on survival. (**A**) Survival curves for glioma patients stratified by mutation count into equal sized groups. **(B)** Mutation count decreases as TET1 expression increases. **(C)** Correlation of predicted gene expression to the TET1 affected genes model predicted hazard. Light blue indicates a positive correlation (increased predicted expression increases predicted hazard); dark blue indicates a negative correlation. **(D)** A scratch test measures cell motility in control and TET1 knockdown U87 cells. The red lines indicate the initial cell-free area. The chart indicates the median wound area in 3–6 experiments at each time point *P < 0.05; **P < 0.01; ***P < 0.001.

Poorer survival is often associated with tumors with more deletions, mutations, and copy number variations^20^. Indeed, we found that glioma patients with a low mutation count (derived from GDC MAF files) had the highest survival probability, followed by moderate and then high mutation counts (Fig. 4A). At 1,000 days as evaluated from day 0 tumor biopsy, the survival probabilities for patients with low, moderate, and high mutation count were approximately 0.8, 0.4, and 0.2, respectively.

If DNA repair mediates the effect of TET1 on survival, we would expect to observe a higher number of mutations in tumors expressing low levels of TET1. The number of mutations drops significantly as TET1 expression increases (Fig. 4B). A driver of mutation count could be inactivation of DNA repair genes.

### Predicted gene expression and direct effect model hazard

We evaluated the contribution of specific genes to the observed percent mediation (Fig. 4C). In DNA repair, negative Pearson correlations were detected in NEIL (nucleotide excision repair)^21^ and in RAD17 (DNA-damage induced cell cycle checkpoint)^22^. Positive Pearson coefficients were found in several genes, including XAB2 metaphase delay^23^, UVRAG (pro autophagy and NOTCH positive regulator)^24^, and SFPQ, a multifunctional pro DNA repair gene^25^, which is also involved in the regulation of L1 retrotransposons and proliferation^26^. Two other genes in the DNA repair hallmark having positive Pearson coefficients include RAD23B (nucleotide excision repair)^27^, and ATXN3 (represses the tumor suppressor PTEN), which is associated with tumor proliferation in gastric, lung, and testicular cancers^28,29^.

The immune response to tumors involves different pathways that stimulate as well as suppress tumor rejection. A strong negative Pearson correlation was determined between IL9/NFKB expression and the direct effect model hazard values (Fig. 4C). IL9 is a cytokine released from the CD4 subset of Th9 cells that is involved in the anti-tumor response^30^. Several positive correlations were observed, including genes in the innate response that included oncostatin M (OSM), a member of the IL-6 family^31^, and the tumor necrosis factor family member TNFSF13 (also referred to as APRIL). Increases in expression of inflammatory genes often promote cancers^32^. VTCN1 is a B7 co-stimulatory family member that blocks antigen presentation^33^. Aggressive gliomas are more difficult to treat because cells are mobile and infiltrate/invade surrounding tissue^34,35^.

The inverse association between TET1 hazard and cell motility was surprising. Positive coefficients were determined between the HMG-CoA reductase, which is the rate-limiting enzyme for cholesterol synthesis, sonic hedgehog (SHH), peroxisomal Biogenesis Factor 5, and the sodium channel epithelium1 beta. Negative associations were determined between the SHH transducer smoothened/frizzled class receptor (Smo), Tyrosine 3- Monooxygenase/Tryptophan 5-Monooxygenase (YWHAE), the RHO family GTPase Vav3, the scavenger receptor CD^36^ (SCARB1), and Coro1A, which belongs to a family of evolutionary conserved actin binding proteins. Interestingly, the Pearson correlation for two genes in the SHH pathway, SHH and Smo, are in opposite directions.

### TET1-deficient cells display poor cell motility

Other studies examining the GDC database reported better migration correlated with poorer survival in patients with gliomas^35^. To assess the causal effects of TET1 deficiency on cell motility, migration was compared between a TET1-deficient and control A172 glioma cell line using the wound-healing assay. TET1-deficient and control cells lines were established as previously described^6,8^. At four hours after initializing migration with the scratch, the control cells migrated considerably into the cell-free area and at 12 hours the control cells were found throughout the area. In contrast, the TET1-deficient A172 glioma cell line did not display significant migration at four hours after initializing migration, and at 12 hours migration was observed but significantly differently compared to the control cell line (Fig. 4D and Supplement Table 2). Similar findings were made when the experiment was repeated with the U87 cell line (data not shown).

## Discussion

This study was conducted to test the hypothesis that genes involved in the DDR mediate the poor prognosis of glioma patients with low levels of TET1. The hypothesis was based on our experimental findings that knocking down TET1 expression increased DNA strand breaks, attenuated cell cycle checkpoint, and increased resistance to ionizing radiation in glioma cell lines^6,8^. We confirmed studies by others of the relationship between poorer survival and low levels of TET1 expression in glioma patients. We further found that the relationship was strongest between TET1 expression and survival in low-grade gliomas. To examine the involvement of the DDR using observational data, we took a novel approach linking neural networks to build mediation evidence. This approach helps us better understand causal relationships.

The DDR protects the integrity of the genome. We presented evidence that the integrity of the genome is compromised when TET1 levels are low. Higher numbers of mutations were found in gliomas with low levels of TET1 expression. Genomic instability increases heterogeneity in the tumor cell population, which has been shown to increase the resistance of tumors to therapy^36,37^. The mechanism for the genomic instability appears to be genes involved in the DDR. A positive Pearson correlation coefficient was determined with RAD23B, but a negative coefficient was observed with NEIL1. Both genes are involved in nucleotide exchange repair. RAD23B recognizes the damage that is excised by NEIL repair^21^. In the absence of NEIL activity, nuclear exchange repair would not be completed even though there is recognition of the damage. The DDR prevents the proliferation of cells that harbor an unstable genome. In a previous study, we found that cycle checkpoints attenuated in TET1-deficient gliomas. In this study, our analysis indicates that genes needed to prevent cell cycle progression are repressed. The decrease in RAD17 removes a G1/S checkpoint^22^. The increases in expression of ATXN3 would be expected to repress the tumor suppressor PTEN, which negates several pathways involved in tumor growth. Indeed, ATXN3 expression is associated with tumor proliferation in gastric, lung, and testicular cancers^28,29^.

We also presented evidence suggesting the involvement of the immune response for mediating poor survival among patients with low levels of TET1 expression. Finding the involvement of the immune response is an advantage of our approach because human immune responses are difficult to model experimentally. Interestingly, differences were observed in the involvement of the innate and acquired immune responses. Two cytokine genes involved in innate immune response, TNFSF13 and OSM, were positively correlated with the direct TET1 associated hazard. This indicates an active innate response. VTCN1 (B7-H4) expression was also positively correlated with the direct TET1 associated hazard. B7-H4 is a member of the co-stimulatory and co-inhibitory B7 family and inhibits T-cell activation and clonal expansion of CD4 and CD8 cells in several different types of tumors, including gliomas^38^. Increases in B7-H4 would attenuate the presentation of glioma antigens and result in a poor immune response. The prognostic value of elevated levels of B7-H4 expression has been reported in gastric^39,40^, pancreatic^40^, and non-small-cell lung cancers^41,42^. Expression positively associates with progression in prostate cancer^43^. One consequence of poor antigen presentation would be the absence of the involvement of Th9 cells, which is a subset of CD4 cells that is involved in anti-cancer immunity^44^. IL-9 expression was negatively correlated with the direct TET1 associated hazard. IL-9 is the cytokine released from Th9 cells. Several experimental models have shown the involvement of Th9 cells in the immune response against tumors. IL-9 was shown to increase expression of p21 and TRAIL in melanoma and also enhances the anti-tumor activity of mast cells^45^. Overall, it appears that the TET1 associated hazard is due to a heightened innate immune response that fails to convert to an effective acquired immune response.

The cell migration pathway was negatively associated with the direct TET1 associated hazard. Examining the contribution of specific genes, positive Pearson correlations were determined with two genes that have been reported to promote migration in gliomas, HMG-CoA reductase^46^ and sonic hedgehog^47^^,^ but negative associations were determined with smoothened/frizzled class receptor (Smo)^48^ and Vav3. Smo48 and Vav3^49^ are transducers and are required to activate intracellular pathways for migration in gliomas. It appears that migration is being induced through extracellular stimulation but that the direct TET1 associated hazard is associated with the expression of genes involved in the intracellular signal pathways required for migration.

While far from a complete framework, the neural network linkage mediation experiment described in this paper is a novel approach to collecting evidence for mediation relationships. The model provides prognostic capacity as well as mediation explanations that can be used to design interventions. Indeed, immune response involvement is difficult to model in humans and thus requires mouse models. Considering the successes in immune modulation for treating cancers, TET1 is a potential marker for predicting whether the gliomas would be responsive to biological agents that target Il-9 and VTCN1.

## Materials/Methods

### Data used for analysis

Three datasets were used: The genomic data commons for glioma patient data; an *in-vitro* TET1 knockdown experiment provided in the Supplemental Table 1; and cancer hallmark gene sets previously reported^18^. All patient and molecular data was aggregated from the Genomic Data Commons (GDC). We built and used an open-source Scala client, GDC-Scala, for the GDC API to collect all data sets. Tutorials for using GDC-Scala are available at the repository address. Data for 703 patients with glioblastoma (GBM) and all other lower-grade gliomas (LGG) was collected. Three kinds of patient data were collected.

1. **Clinical data:** Patient days-to-death and days-to-last-follow-up were collected from GDC clinical xml files. These files additionally provide histological data. See GDC documents on clinical data harmonization: gdc.cancer.gov/about-data/data-harmonization-and-generation/clinical-data-harmonization
2. **Expression data:** RNA-seq data was collected for each patient. Data was log normalized and expression was scaled to standard normal. We used the UQ-FPKM gene expression quantification. See GDC documents on transcriptome profiling: gdc.cancer.gov/about-data/data-harmonization-and-generation/genomic-data-harmonization/high-level-data-generation/rna-seq-quantification
3. **Mutation data:** MAF files for glioma patients were collected from the GDC. Total mutation counts were aggregated for each patient. See GDC documents on MAF files: docs.gdc.cancer.gov/Data/File_Formats/MAF_Format/

Code for processing this data can be carefully reviewed at the GDC-scala open source repository available at bitbucket.org/insilica/gdc-scala). All data was collected between July and August 2018.

#### TET1 gene expression knockdown experiment

Data from a TET1 knockdown experiment is provided (Supplemental Table 1). This dataset consists of gene expression signal values for the TET1 knockdown and control A172 glioma cell lines^6^. A fold change is calculated by shEV gMeanSignal/shTET1_rMeanSignal. The table has 44,496 rows. Gene names were mapped to ensembl identifiers using BiomaRt^50^. Genes could have repeated probes.

#### TET1 cell motility knockdown experiment

TET1 knockdown and control cell lines were established as previously described and plated in 6-well tissue culture treated plates at 600,000 cells per well^8^. When cells were 80% confluent, a scratch was made with a P1000 pipet tip and the cell-free area (wound) was imaged using a 20X objective (time 0). The wounds were then tracked and imaged at the times reported. Migration was quantified by calculating the area without cells normalized to the area of the wound at time 0 using Image J. Average relative area of the wound was calculated over 3–6 images per time point.

#### Hallmark gene sets

Cancer hallmark gene sets were selected as described originally in colorectal cancer to find sets of prognostic hallmark-based gene signatures^18^. These gene sets are a good selection because of their prognostic relevance and their membership in important cancer hallmark gene sets. We selected eight gene sets (DNA Repair, Immune Response, Phos1, Phos2, Cell Motility, Cell Cycle, Cell Death, and Apoptosis). These gene sets were reduced to 10–20 genes by removing genes without complete expression data across all glioma patients.

#### Model creation

Several models were created in this study. Cox proportional hazards models and Kaplan-Meier curves were created in R. Two classes of neural network models were created: Survival Neural Networks and Multitask Neural Networks. Every model is created and evaluated in cross-validation, including the concordance and percent mediated effect values.

#### Survival networks

The SurvivalNet package was used to build neural networks that estimate patient hazard from gene expression^16^. These networks combine normal deep learning networks with a Cox proportional hazards output layer and loss function. The gene expression inputs were always log transformed and standard scalar normalized. The output is patient hazard with a loss function derived from days-to-death and days-to-last-follow-up. The models include the following: (1) *TET1 only model:* A survival network with TET1 expression input and patient hazard output; (2) *TET1 affected genes model:* A model with 12 inputs including TET1 expression and expression of genes with high fold change in a TET1 knockdown experiment. This model estimates patient hazard as a function of TET1 and its differentially expressed genes; (3) *Hallmark models:* Eight survival networks using expression of genes from the selected hallmark; and (4) *Permutation models:* 50 survival networks built from random selections of 10 genes.

The neural network architectures were determined by Bayesian optimization, for the task of selecting the following proper hyperparameters: dropout rate, number of nodes in the single hidden layer, batch size, and number of epochs.

#### Multitask Networks

The Keras Python package (https://keras.io/) was used for construction of multitask neural networks. These multitask networks (one constructed for each hallmark gene set and permutation set) use log transformed expression of TET1 and its affected genes as inputs, feed-forward hidden layers with rectified linear (ReLU) activations of size 100, and a regression output for each log transformed expression of genes in respective gene sets. An ADAM optimizer was used.

### Model concordance

Survival models were evaluated by measuring their patient concordance. Each survival model generates a numeric hazard value for each patient. Pairs of patients can then be ordered by this hazard value. A concordant count is derived from all the pairs of patients where the patient with a higher hazard value is known to have died before the patient with a lower survival value. The discordant count tracks all pairs of patients where the patient with higher hazard is known to have died last. The concordance value is then the concordant count divided by the total (discordant + concordant). Concordance thus partially tracks pairs of patients where one is lost to follow-up.

#### Mediation experiment

The objective of the mediation experiments was to investigate pathways mediating the TET1 effect on survival and not to build the strongest possible glioma survival model. Mediation is determined by inputting predicted expression values from multitask models into the pre-trained hallmark survival networks. The concordance of this composed model is then computed on the set of patient pairs ranked correctly by the TET1 and affected genes’ survival network. This concordance is reported as the percent mediated effect. Our rationale is that the percent mediated effect does not take into account the mediating models performance on pairs of patients incorrectly predicted by the TET1 and affected genes’ survival network. This is because patients incorrectly predicted by the base model who are correctly predicted after mediation experiment tell us nothing about mediation of the base model effect. The percent mediated effect improves upon simply measuring correlation between the hallmark predicted hazard and the base model predicted hazard because it requires that hallmark gene expression be predicted from base network gene expression. This approach is motivated by traditional mediation analysis^51,52^.

#### Code

Using the R code can reproduce this project, including all figures and analyses and Python code available in the repository. https://bitbucket.org/insilica/tet1survival.

#### Packages

The below is a list of the analytics packages used in this work:

#### Python

- **SurvivalNet:** A Cox proportional hazards output layer is added to a traditional neural network. This output layer allows network parameters to be fit based on errors derived from survival data. SurvivalNet models are built for (1) TET1 + TET1 affected genes, and (2) all gene set hallmarks. See https://github.com/CancerDataScience/SurvivalNet
- **Keras:** Keras is a deep learning package written for Python. We use this package to build multitask neural networks. These multitask networks have multiple outputs corresponding to expression of different genes.

#### **R**

- **ggsignif**: Significance calculation and box plots: https://github.com/const-ae/ggsignif
- **survminer:** Survival curve visualization: https://github.com/kassambara/survminer
- **survival:** Basic survival analytics (survival functions, coxph, etc.)
- **gplots:** Hallmark correlation heatmaps^53^.

#### Scala

- **Apache Spark:** Used for processing of expression, mutation, and clinical patient files
- **GDC-Scala:** An open-source client for the Genomic Data Commons API (https://bitbucket.org/insilica/gdc-scala/src/master/).

## Supplementary information

Supplementary information

Supplemental data set.
