# Supplementary material for: Measuring Cancer Hallmark Mediation of the TET1 Glioma Survival Effect with Linked Neural-Network Based Mediation Experiments
Source: Sci Rep. 2020 Jun 1;10:8886. doi: 10.1038/s41598-020-65369-3 (PMC7264360; doi:10.1038/s41598-020-65369-3)
Supplement: Supplementary file 1 — Supplementary information [file 41598_2020_65369_MOESM1_ESM.pdf]

Supplementary Data

Measuring Cancer Hallmark Mediation of the TET1 Glioma Survival Effect with Linked Neural-Network Based Mediation Experiments.

Thomas Luechtefeld[1], Nole Lin[1], Channing Paller[2], Katherine Kuhns[3], John J. Laterra[2,4], Joseph P. Bressler[3,4*]

Affiliations:

1. Insilica LLC, 2736 Quarry Heights Way, Baltimore, MD, USA
2. Johns Hopkins Sidney Kimmel Comprehensive Cancer Center, Baltimore, MD, USA
3. Department of Environmental Health and Engineering, Bloomberg School of Public Health, Johns Hopkins University, Baltimore, MD, USA
4. Kennedy-Krieger Institute, Baltimore, MD, USA

***Correspondence:**

Address for correspondence: Joseph Bressler, Department of Neurology, Kennedy-Krieger Institute, 707 North Broadway, Baltimore, MD 21205. Phone: 443-923-2677; fax: 443-923-2695.

E-mail address: bressler@kennedykrieger.org

Running Title: TET1 Glioma Survival Effect Model

Key Words: Neural Network, Cancer Hallmark, TET1, Glioma, Survival

Supplemental Analysis

Figure 1

Correlations between TET1 and Age, Histology / IDH1

TET1 is differentially expressed with histological type, IDH mutation status and age - factors that all affect survival. This indicates that the risk associated with TET1 may be confounded. Low TET1 (associated with greater hazard) is also associated with greater risk due to age (greater age), greater risk due to histology (TET1 has lower expression in GBM than LGG), and greater risk due to IDH mutation (lower expression in patients without IDH1 mutations).

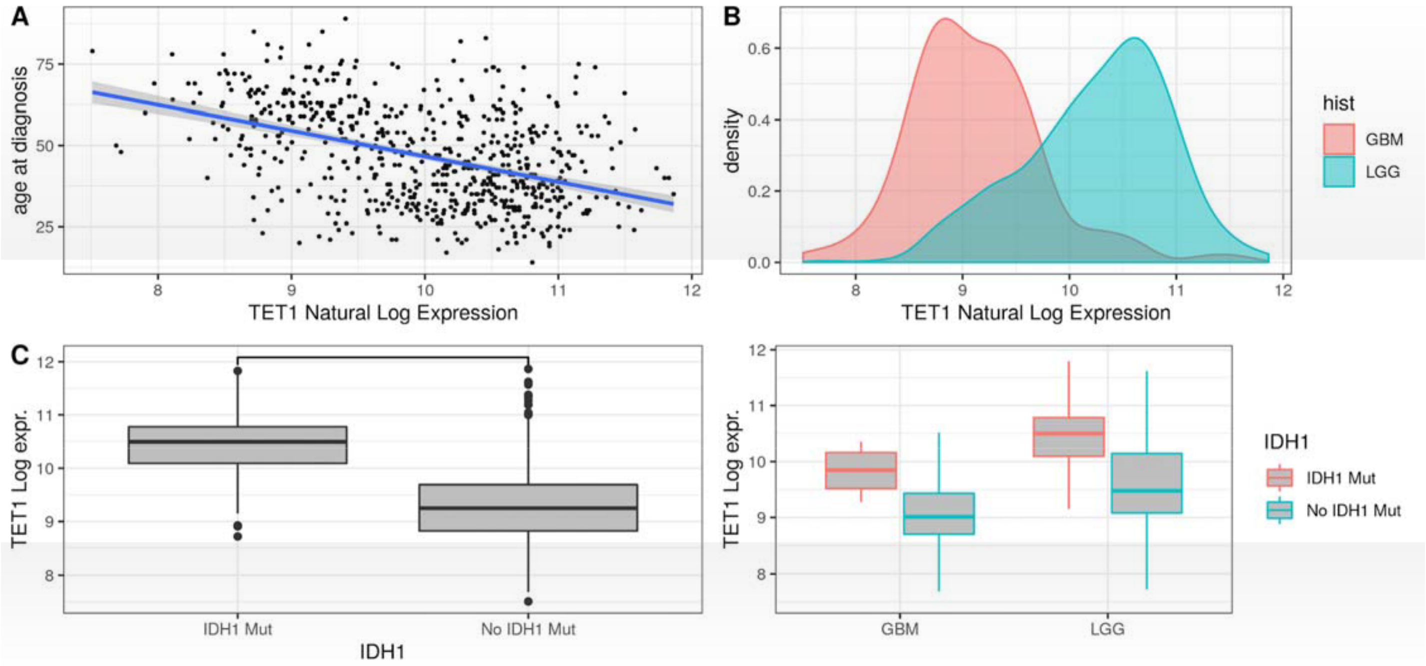

Figure 1 A. TET1 expression increases with decreasing age. **B.** TET1 expression is much higher in LGG than GBM. **C.** TET1 expression is significantly higher in patients with an IDH mutation. **D.** Relative expression of TET1 is increased with IDH1 mutation in both LGG patients and GBM patients

These correlations indicate that the observed TET1 hazard is confounded by age, histology, and IDH1 status. The next sections provide evidence that risk associated with TET1 persists when controlling for these factors.

Figure 2

Multivariate Cox Proportional Hazard Forest Plot

A multivariate cox proportional hazards model was constructed on TET1 expression (Q3/4,Q2,Q1 refer to quartile with Q1 the lowest), age, IDH1 mutation status, and histology to evaluate whether TET1 hazard is confounded by other risk factors.

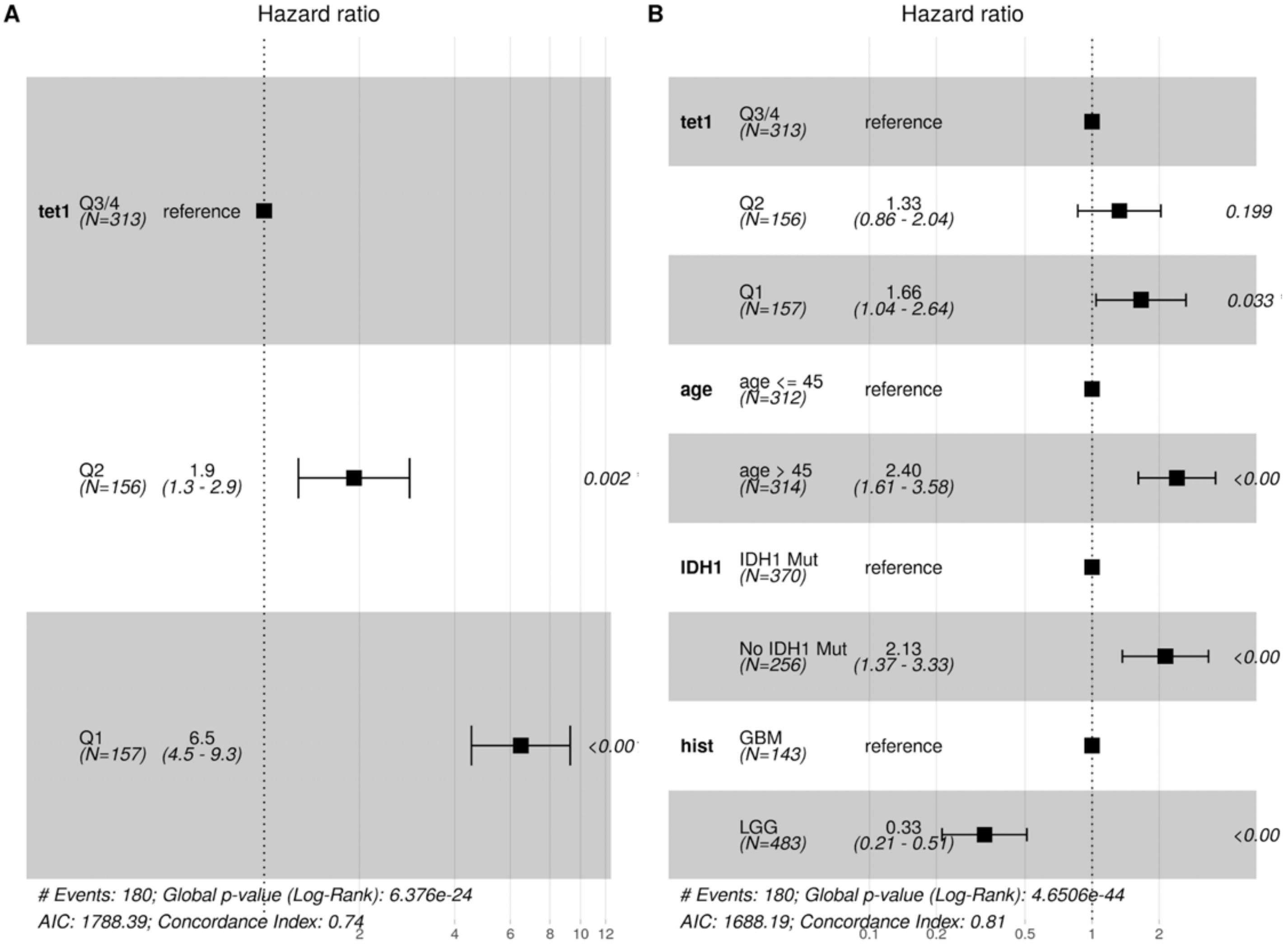

Supplemental Figure 2: (A) Cox model forest plot built on all glioma patients with TET1 quartile expression alone. **(B)** Cox model forest plot built on all glioma patients with survival as a function of TET1 quartile expression + age + IDH1 + histology.

The lowest quartile of TET1 expression is significantly associated with glioma hazard in a multivariate model of TET1 + age + IDH1 status + Histological type. The reduction in TET1 significance in the multivariate (right) relative to the univariate model (left) indicates partial dependence of TET1 survival effect on these factors.

Figure 3

Univariate Patient Set Forest Plots (Figure 1B supplement)

The below 6 forest plots track the significance and confidence intervals of TET1 hazard intervals from the 6 kaplan meier curve plots in Figure 1 of the publication. Expression quartiles are derived from the full patient set and not for each set of patients. The tet1 hazard ratio is significant at the 0.001 level for at least the lowest quartile of expression in all categories except GBM patients.

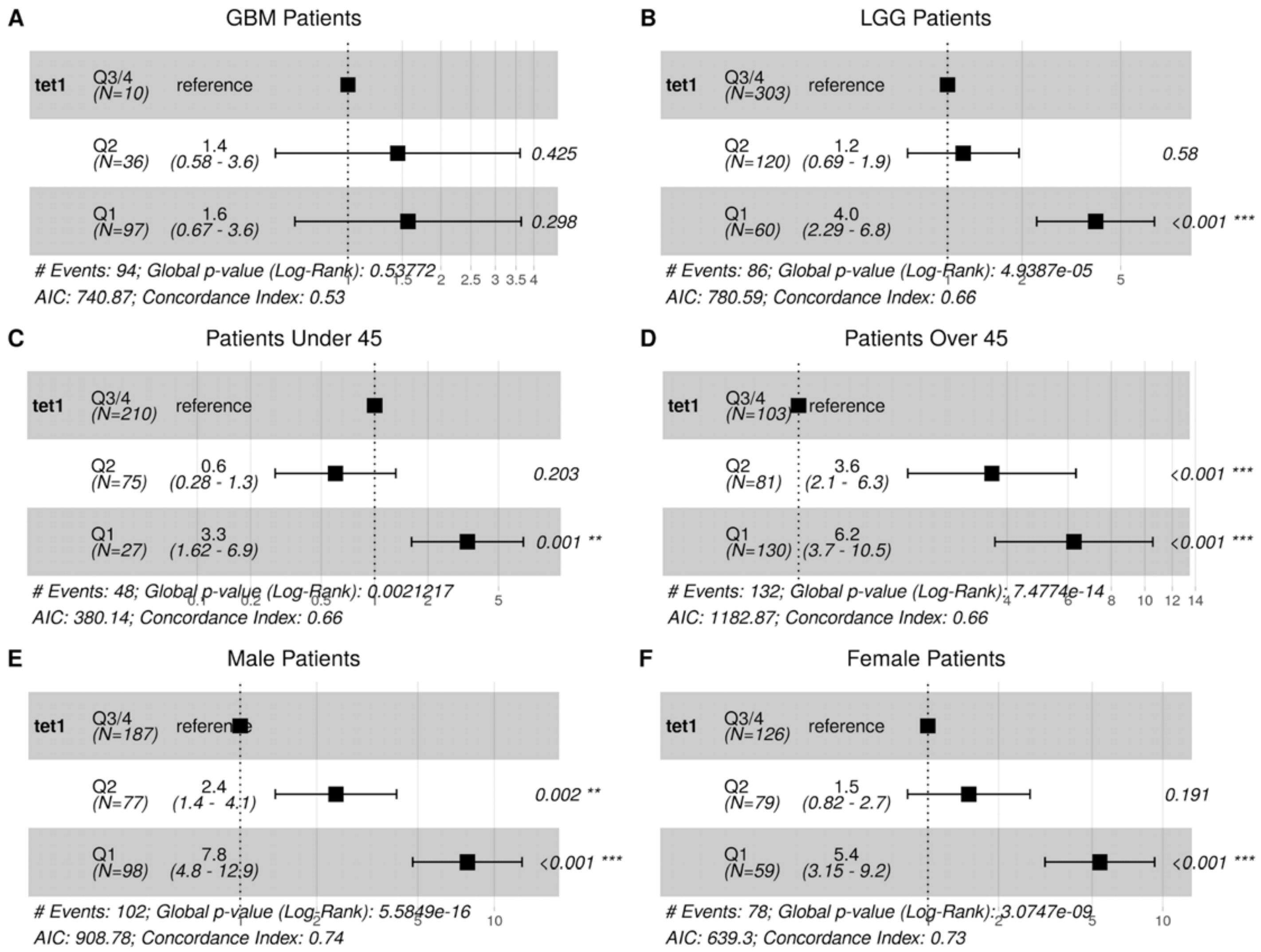

Figure 4

Multivariate Patient Set Forest Plots (Figure 1B supplement)

To further investigate whether low TET1 expression remains a risk factor after accounting for potential confounders, we reproduce the patient set specific forest plots from the last section, but use multivariate cox proportional hazards models.

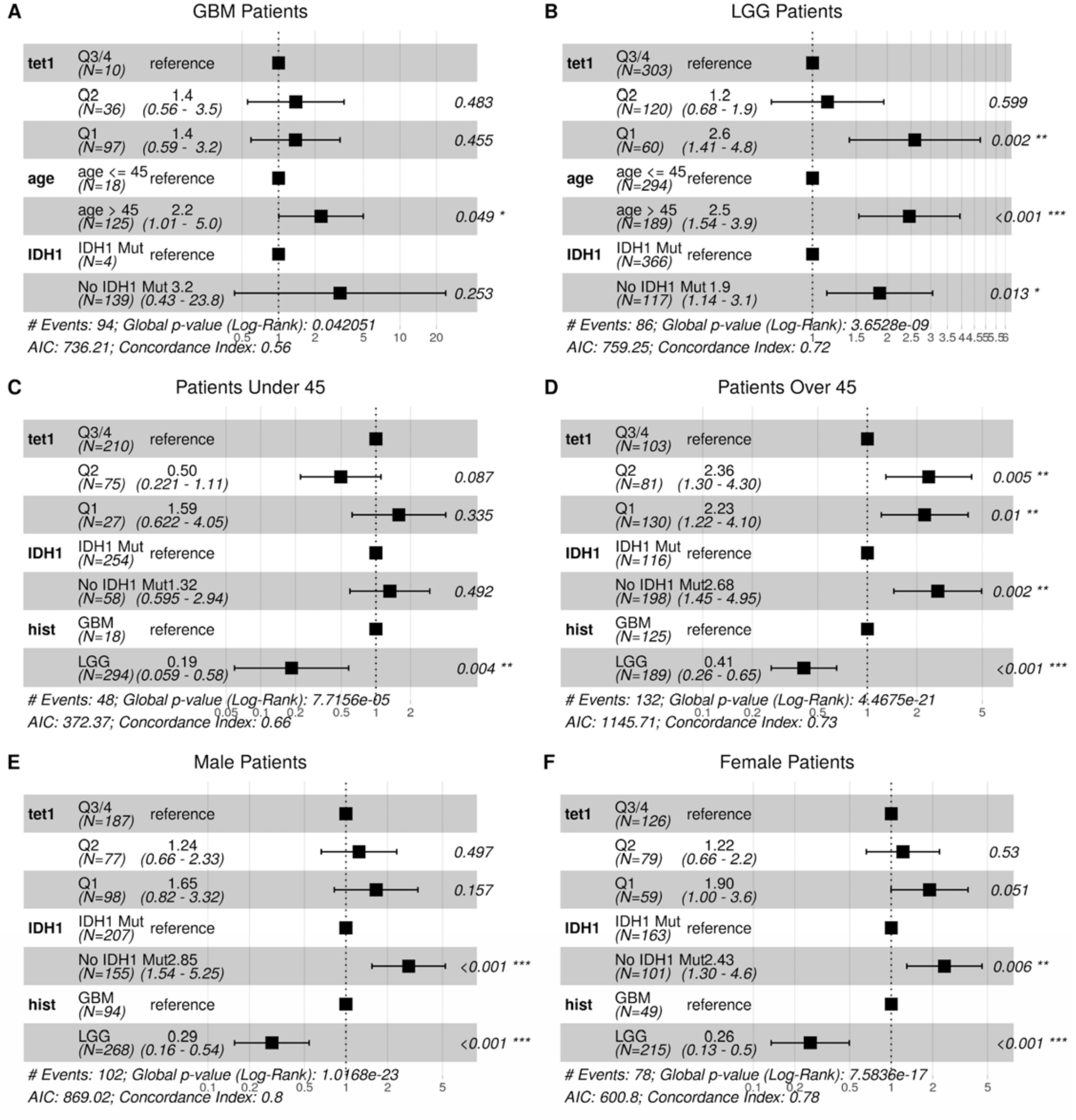

In these patient set specific multivariate cox models- low TET1 expression (Q1) remains trending towards greater hazard or significantly associated with greater hazard.

Table 2 Scratch Assay Results

Figure 4 in the publication presents a chart and image from a TET1 knockdown scratch test. Here we present some of the numeric data generated in a set of scratch test experiments.

**Hours**	**Control (+/- S.E.M)**	**ShTET1 (+/- S.E.M)**
4	0.76 (0.1)	0.91 (0.15)
8	0.32 (0.02)**	0.74 (0.1)*
12	0.10 (0.002)***	0.64 (0.11)*

Scratch assay numeric results from Figure 4D.

- *P < 0.05; **P <0.01; ***P < 0.001;
- Table indicates the median wound area in 3-6 experiments at each time point.
- Each value was compared to the initial cell free area

Supplemental Analysis

Figure 1

Correlations between TET1 and Age, Histology / IDH1

TET1 is differentially expressed with histological type, IDH mutation status and age - factors that all affect survival. This indicates that the risk associated with TET1 may be confounded. Low TET1 (associated with greater hazard) is also associated with greater risk due to age (greater age), greater risk due to histology (TET1 has lower expression in GBM than LGG), and greater risk due to IDH mutation (lower expression in patients without IDH1 mutations).

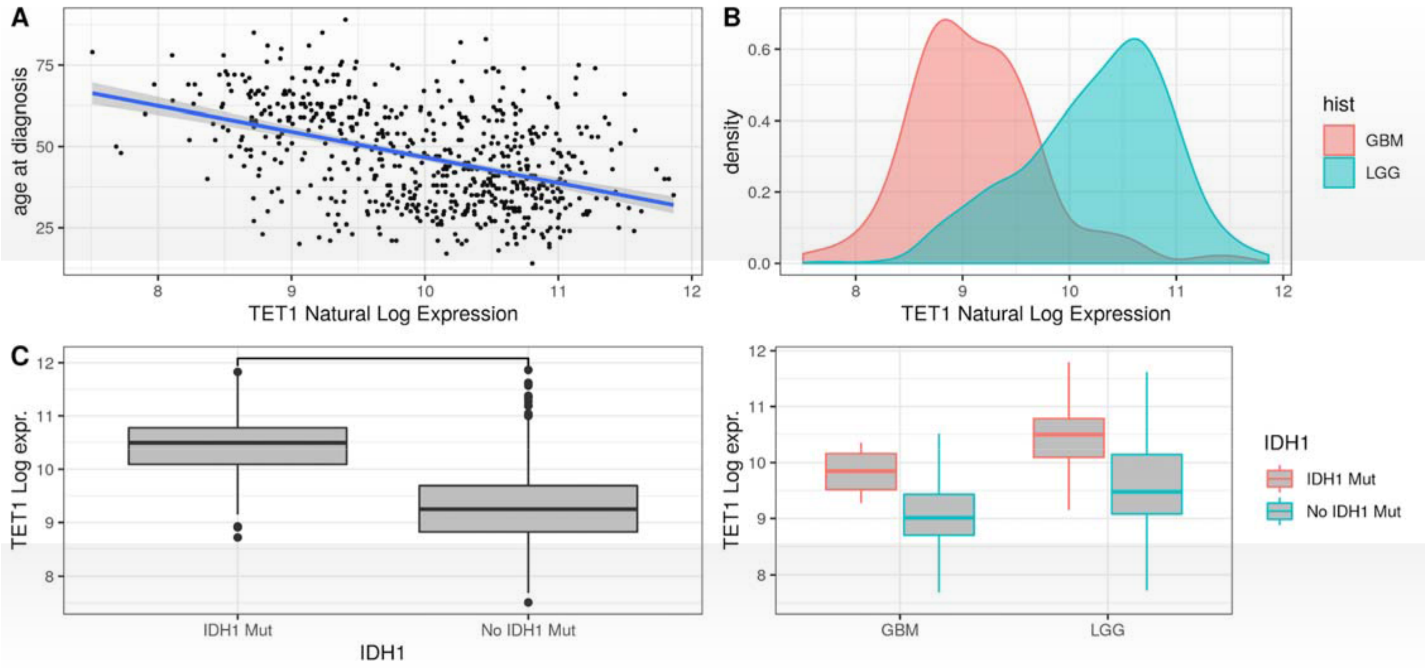

Figure 1 A. TET1 expression increases with decreasing age. **B.** TET1 expression is much higher in LGG than GBM. **C.** TET1 expression is significantly higher in patients with an IDH mutation. **D.** Relative expression of TET1 is increased with IDH1 mutation in both LGG patients and GBM patients

These correlations indicate that the observed TET1 hazard is confounded by age, histology, and IDH1 status. The next sections provide evidence that risk associated with TET1 persists when controlling for these factors.

Figure 2

Multivariate Cox Proportional Hazard Forest Plot

A multivariate cox proportional hazards model was constructed on TET1 expression (Q3/4,Q2,Q1 refer to quartile with Q1 the lowest), age, IDH1 mutation status, and histology to evaluate whether TET1 hazard is confounded by other risk factors.

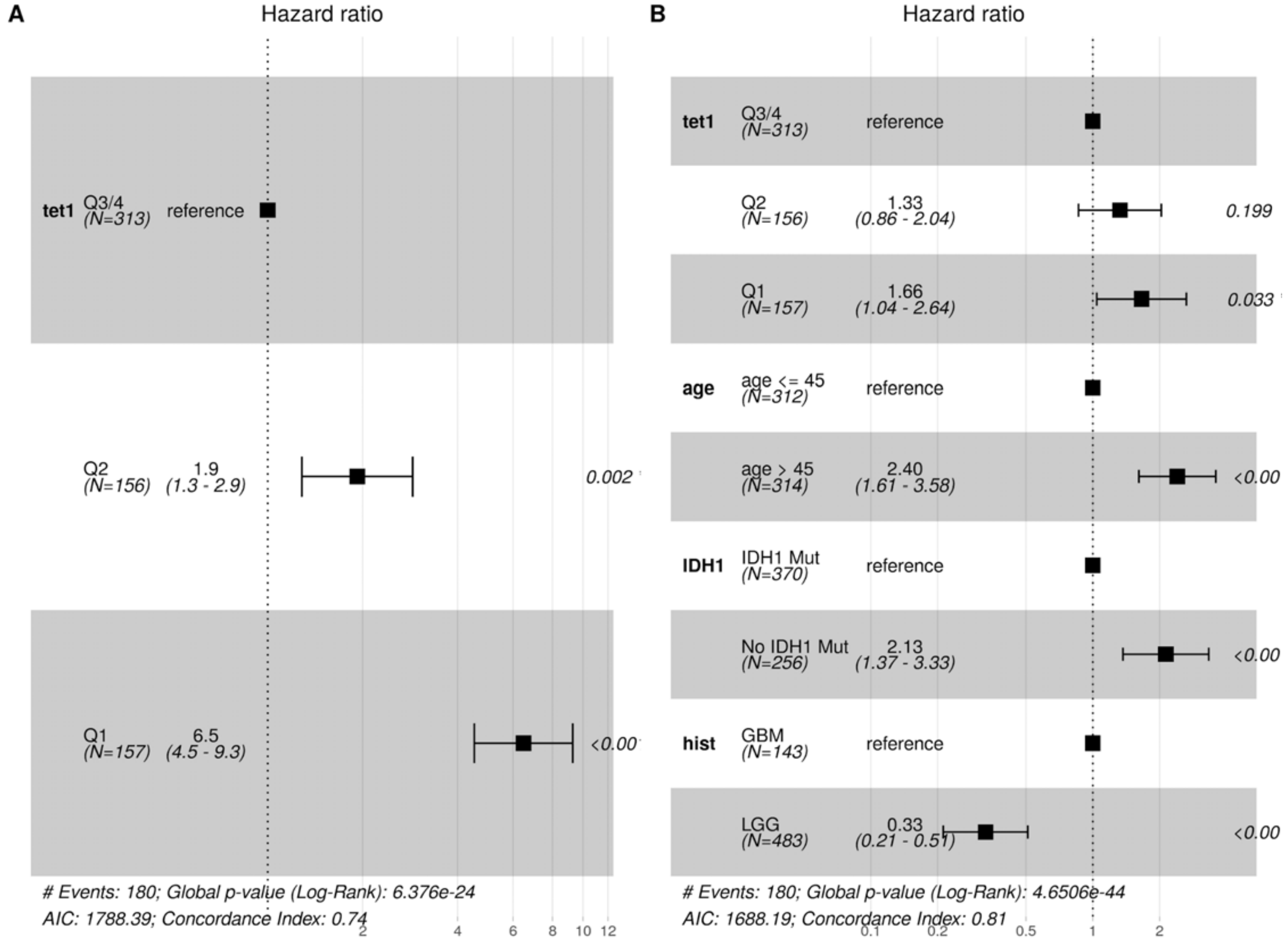

Supplemental Figure 2: (A) Cox model forest plot built on all glioma patients with TET1 quartile expression alone. **(B)** Cox model forest plot built on all glioma patients with survival as a function of TET1 quartile expression + age + IDH1 + histology.

The lowest quartile of TET1 expression is significantly associated with glioma hazard in a multivariate model of TET1 + age + IDH1 status + Histological type. The reduction in TET1 significance in the multivariate (right) relative to the univariate model (left) indicates partial dependence of TET1 survival effect on these factors.

Figure 3

Univariate Patient Set Forest Plots (Figure 1B supplement)

The below 6 forest plots track the significance and confidence intervals of TET1 hazard intervals from the 6 kaplan meier curve plots in Figure 1 of the publication. Expression quartiles are derived from the full patient set and not for each set of patients. The tet1 hazard ratio is significant at the 0.001 level for at least the lowest quartile of expression in all categories except GBM patients.

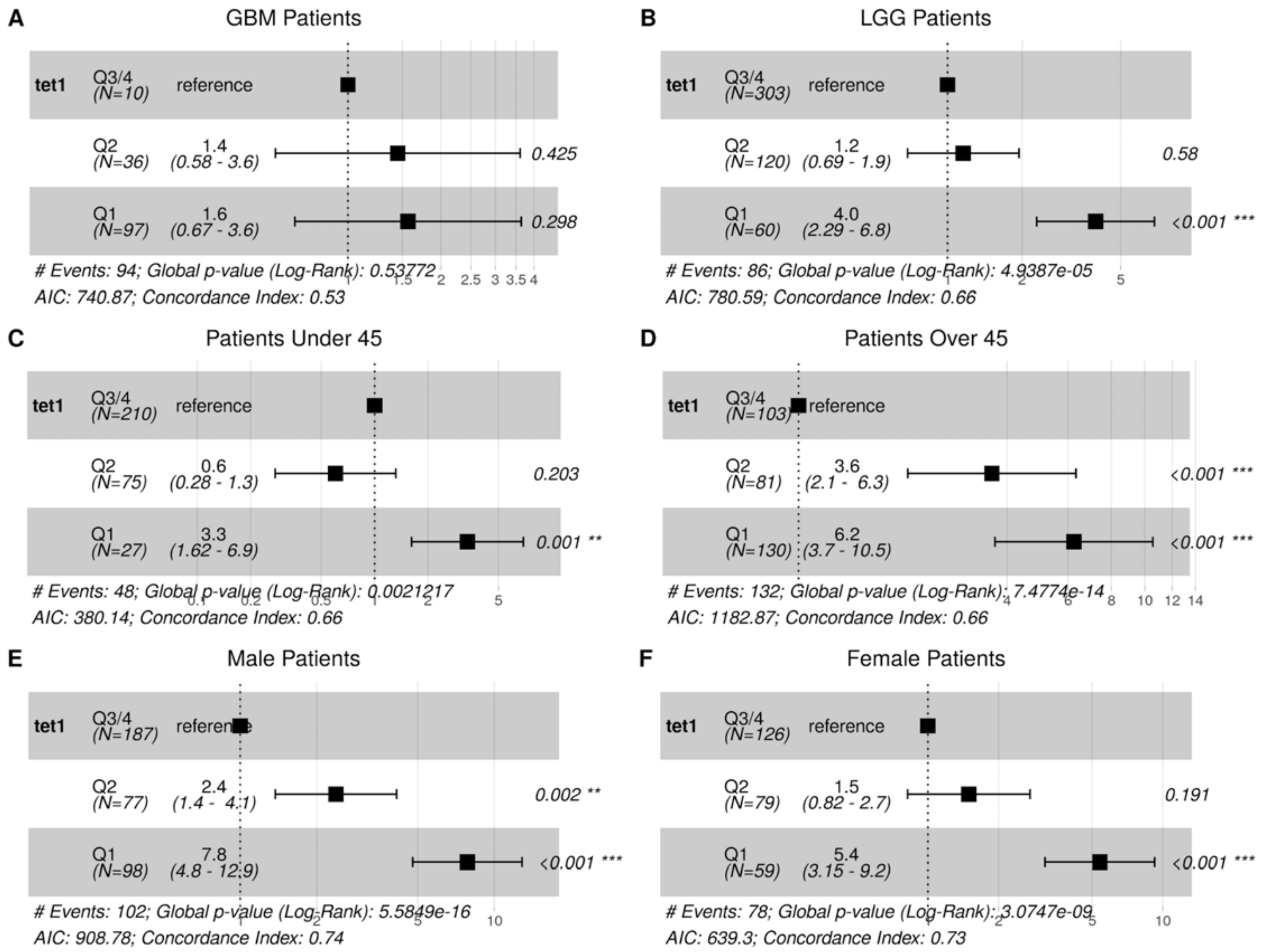

Figure 4

Multivariate Patient Set Forest Plots (Figure 1B supplement)

To further investigate whether low TET1 expression remains a risk factor after accounting for potential confounders, we reproduce the patient set specific forest plots from the last section, but use multivariate cox proportional hazards models.

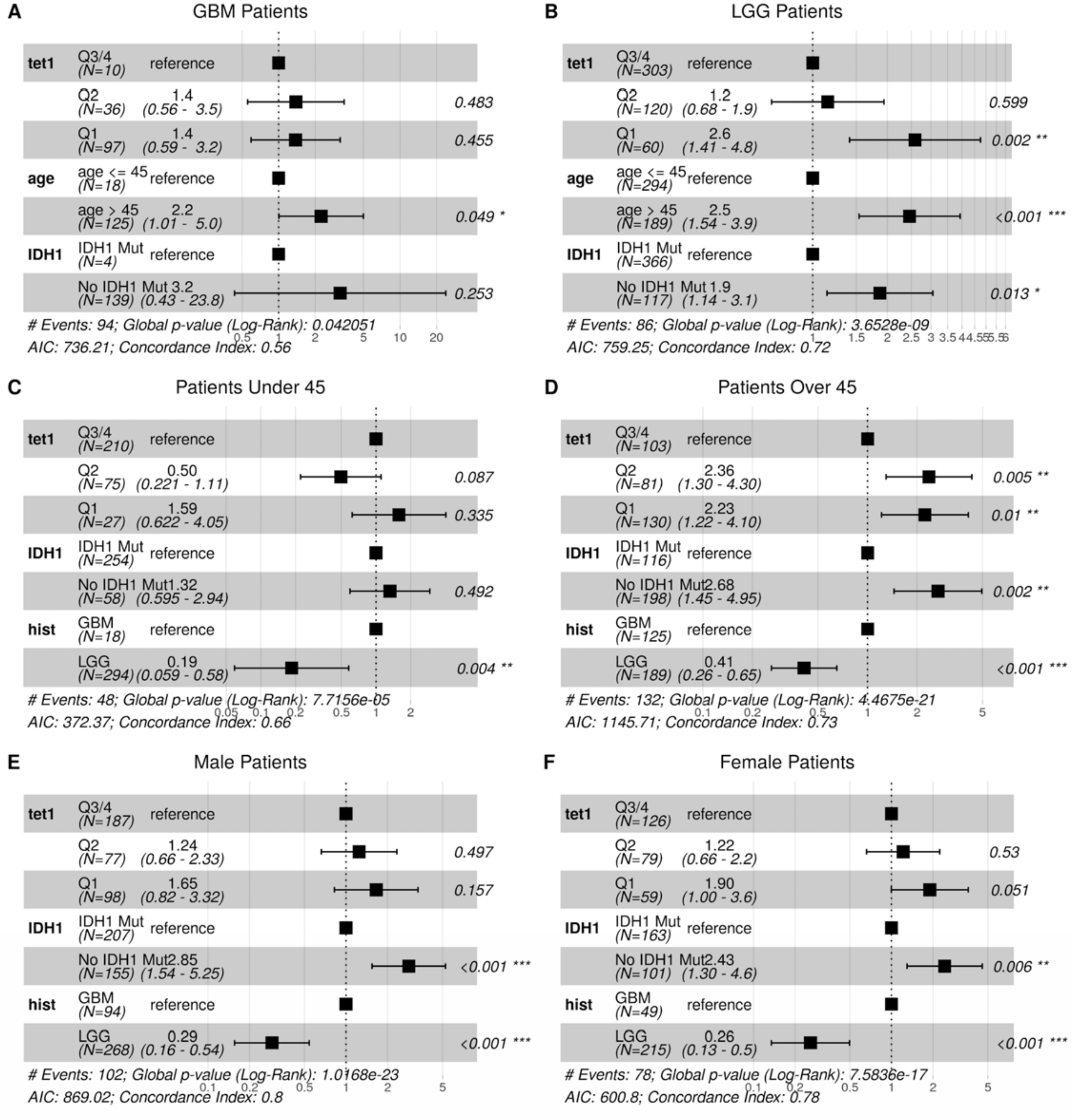

In these patient set specific multivariate cox models- low TET1 expression (Q1) remains trending towards greater hazard or significantly associated with greater hazard.

Table 2 Scratch Assay Results

Figure 4 in the publication presents a chart and image from a TET1 knockdown scratch test. Here we present some of the numeric data generated in a set of scratch test experiments.

**Hours**	**Control (+/- S.E.M)**	**ShTET1 (+/- S.E.M)**
4	0.76 (0.1)	0.91 (0.15)
8	0.32 (0.02)**	0.74 (0.1)*
12	0.10 (0.002)***	0.64 (0.11)*

Scratch assay numeric results from Figure 4D.

- *P < 0.05; **P <0.01; ***P < 0.001;
- Table indicates the median wound area in 3-6 experiments at each time point.
- Each value was compared to the initial cell free area

Supplemental Analysis

Figure 1

Correlations between TET1 and Age, Histology / IDH1

TET1 is differentially expressed with histological type, IDH mutation status and age - factors that all affect survival. This indicates that the risk associated with TET1 may be confounded. Low TET1 (associated with greater hazard) is also associated with greater risk due to age (greater age), greater risk due to histology (TET1 has lower expression in GBM than LGG), and greater risk due to IDH mutation (lower expression in patients without IDH1 mutations).

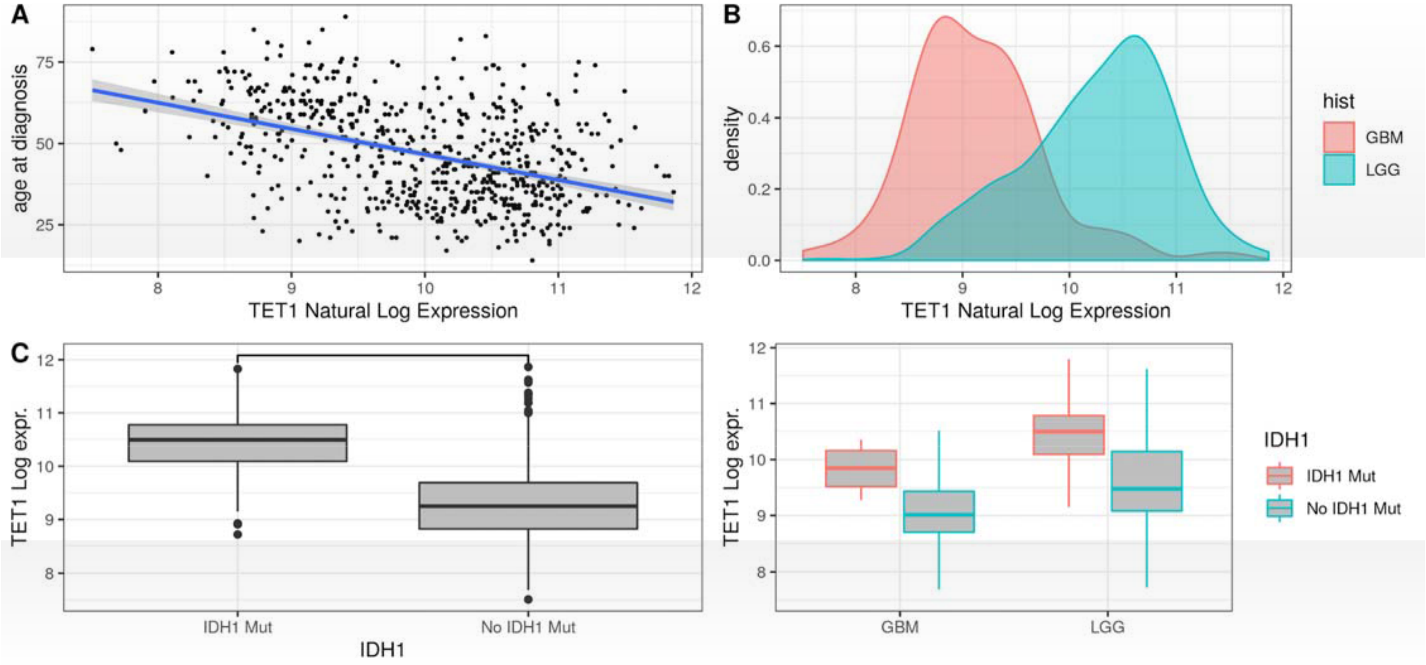

Figure 1 A. TET1 expression increases with decreasing age. **B.** TET1 expression is much higher in LGG than GBM. **C.** TET1 expression is significantly higher in patients with an IDH mutation. **D.** Relative expression of TET1 is increased with IDH1 mutation in both LGG patients and GBM patients

These correlations indicate that the observed TET1 hazard is confounded by age, histology, and IDH1 status. The next sections provide evidence that risk associated with TET1 persists when controlling for these factors.

Figure 2

Multivariate Cox Proportional Hazard Forest Plot

A multivariate cox proportional hazards model was constructed on TET1 expression (Q3/4,Q2,Q1 refer to quartile with Q1 the lowest), age, IDH1 mutation status, and histology to evaluate whether TET1 hazard is confounded by other risk factors.

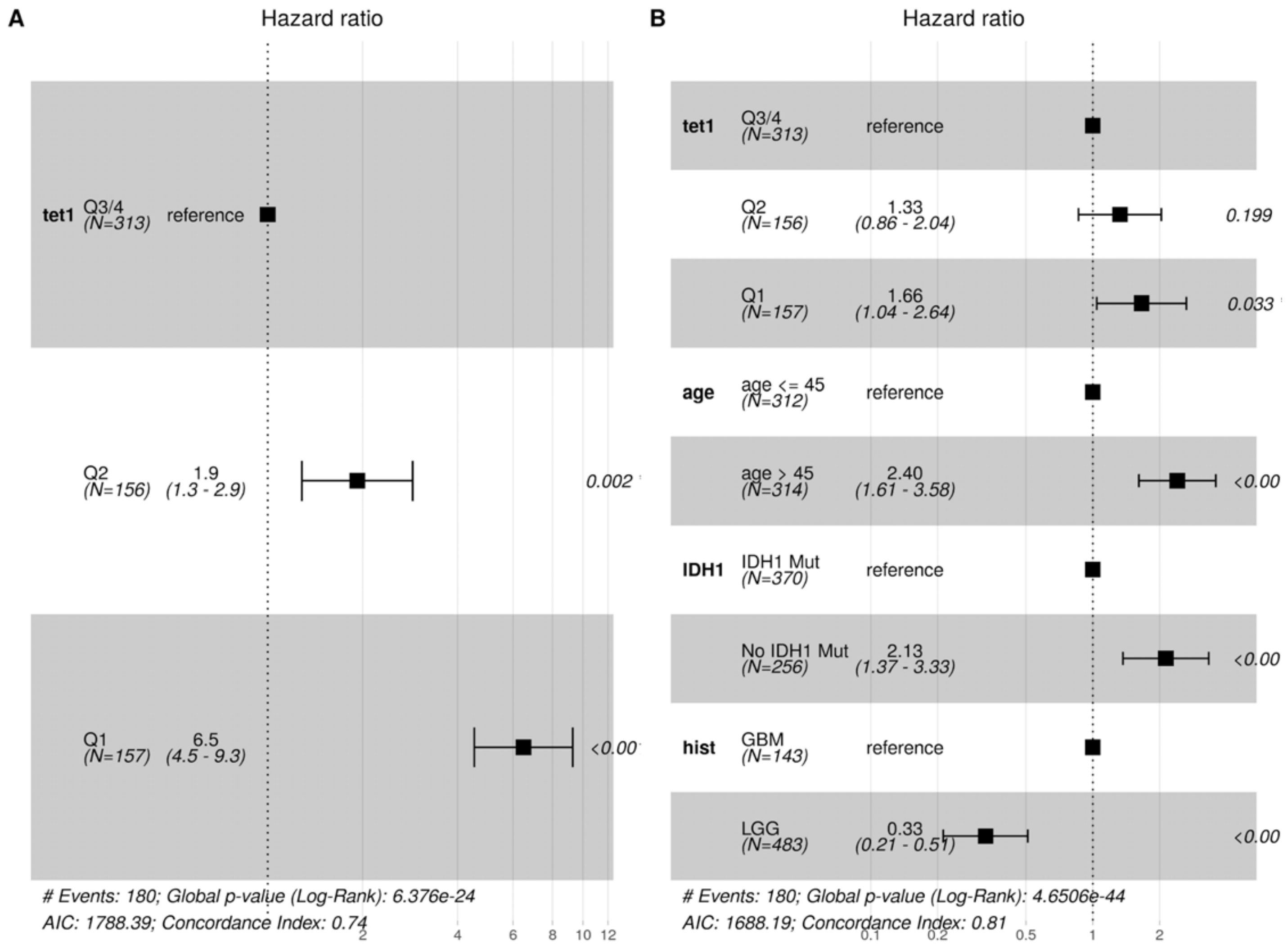

Supplemental Figure 2: (A) Cox model forest plot built on all glioma patients with TET1 quartile expression alone. **(B)** Cox model forest plot built on all glioma patients with survival as a function of TET1 quartile expression + age + IDH1 + histology.

The lowest quartile of TET1 expression is significantly associated with glioma hazard in a multivariate model of TET1 + age + IDH1 status + Histological type. The reduction in TET1 significance in the multivariate (right) relative to the univariate model (left) indicates partial dependence of TET1 survival effect on these factors.

Figure 3

Univariate Patient Set Forest Plots (Figure 1B supplement)

The below 6 forest plots track the significance and confidence intervals of TET1 hazard intervals from the 6 kaplan meier curve plots in Figure 1 of the publication. Expression quartiles are derived from the full patient set and not for each set of patients. The tet1 hazard ratio is significant at the 0.001 level for at least the lowest quartile of expression in all categories except GBM patients.

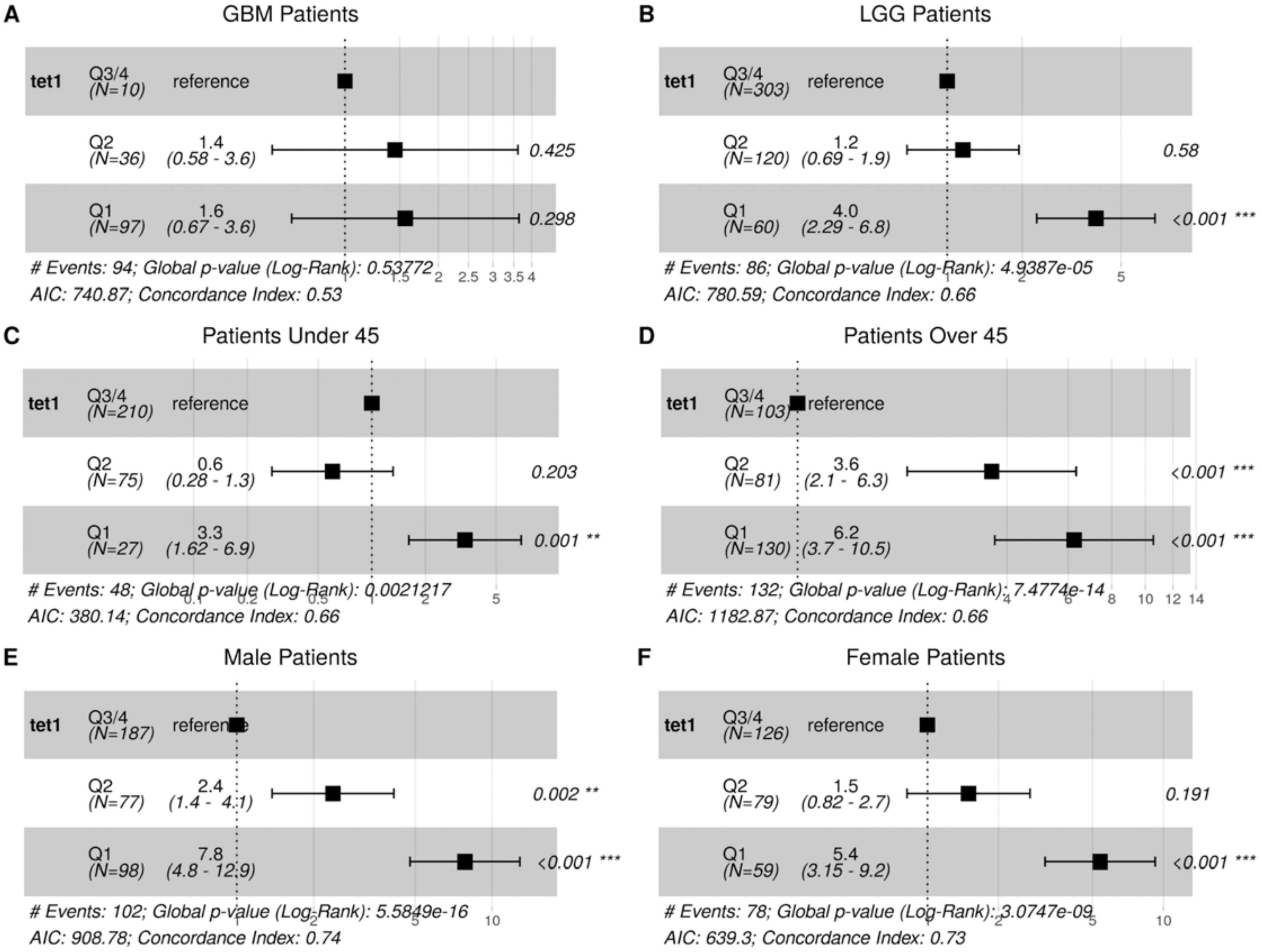

Figure 4

Multivariate Patient Set Forest Plots (Figure 1B supplement)

To further investigate whether low TET1 expression remains a risk factor after accounting for potential confounders, we reproduce the patient set specific forest plots from the last section, but use multivariate cox proportional hazards models.

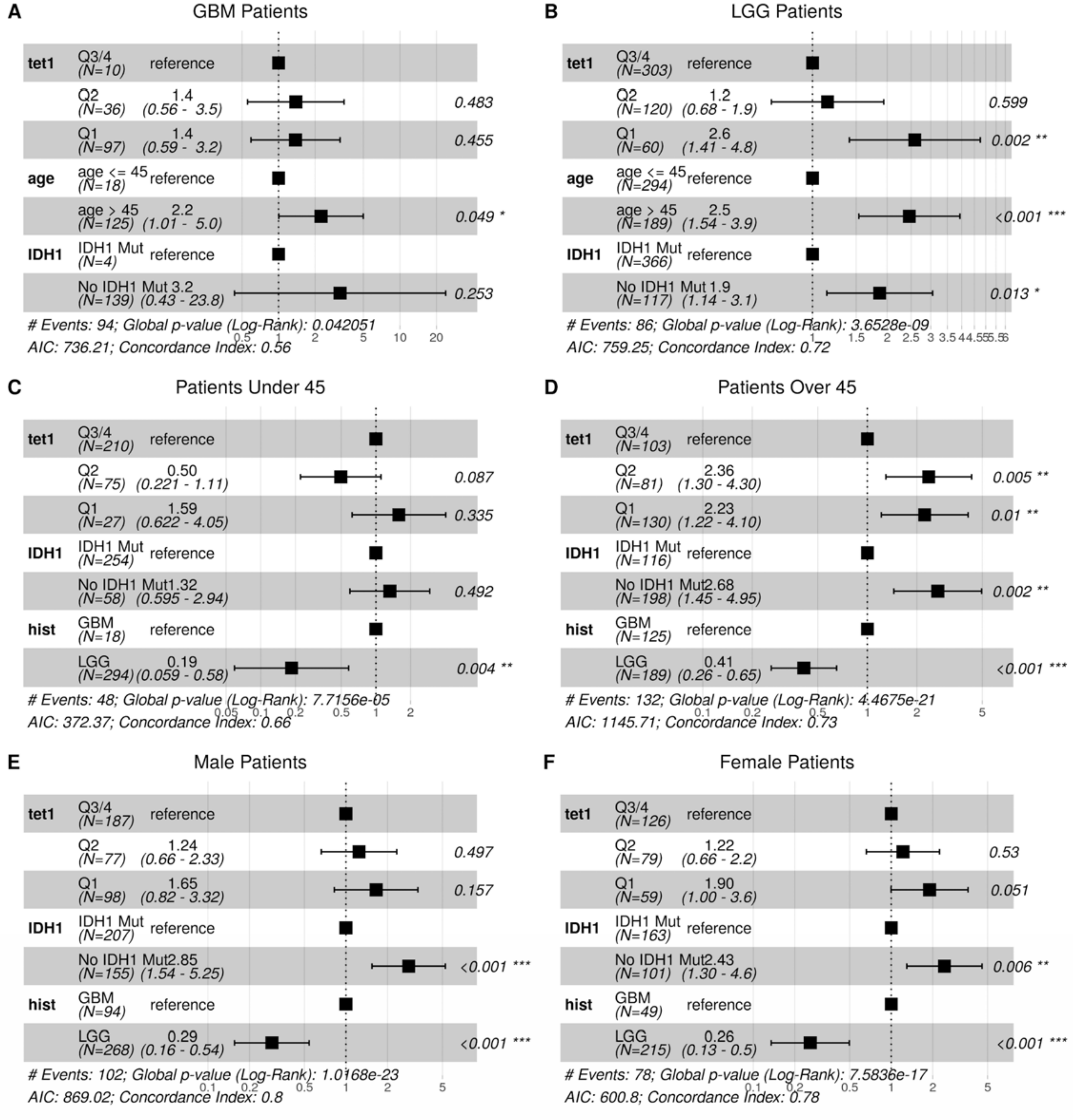

In these patient set specific multivariate cox models- low TET1 expression (Q1) remains trending towards greater hazard or significantly associated with greater hazard.

Table 2 Scratch Assay Results

Figure 4 in the publication presents a chart and image from a TET1 knockdown scratch test. Here we present some of the numeric data generated in a set of scratch test experiments.

**Hours**	**Control (+/- S.E.M)**	**ShTET1 (+/- S.E.M)**
4	0.76 (0.1)	0.91 (0.15)
8	0.32 (0.02)**	0.74 (0.1)*
12	0.10 (0.002)***	0.64 (0.11)*

Scratch assay numeric results from Figure 4D.

- *P < 0.05; **P <0.01; ***P < 0.001;
- Table indicates the median wound area in 3-6 experiments at each time point.
- Each value was compared to the initial cell free area

Supplemental Analysis

Figure 1

Correlations between TET1 and Age, Histology / IDH1

TET1 is differentially expressed with histological type, IDH mutation status and age - factors that all affect survival. This indicates that the risk associated with TET1 may be confounded. Low TET1 (associated with greater hazard) is also associated with greater risk due to age (greater age), greater risk due to histology (TET1 has lower expression in GBM than LGG), and greater risk due to IDH mutation (lower expression in patients without IDH1 mutations).

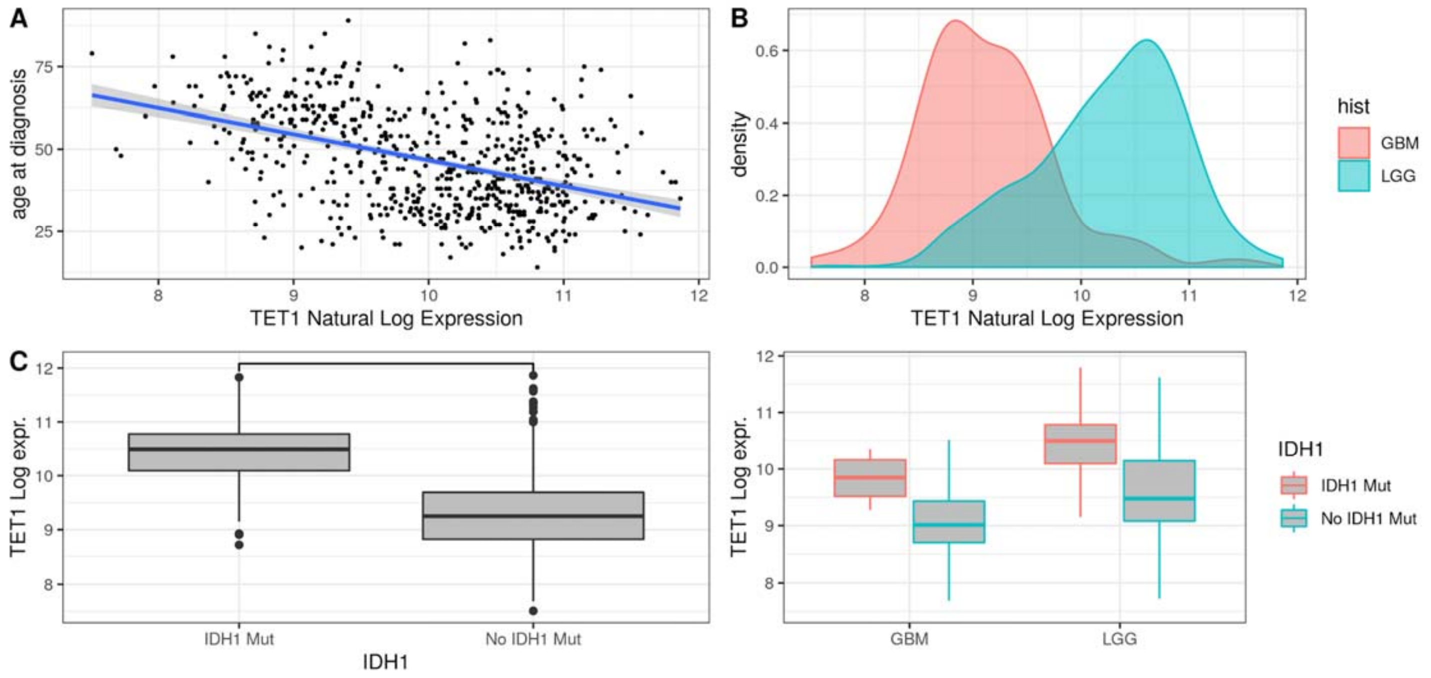

Figure 1 A. TET1 expression increases with decreasing age. **B.** TET1 expression is much higher in LGG than GBM. **C.** TET1 expression is significantly higher in patients with an IDH mutation. **D.** Relative expression of TET1 is increased with IDH1 mutation in both LGG patients and GBM patients

These correlations indicate that the observed TET1 hazard is confounded by age, histology, and IDH1 status. The next sections provide evidence that risk associated with TET1 persists when controlling for these factors.

Figure 2

Multivariate Cox Proportional Hazard Forest Plot

A multivariate cox proportional hazards model was constructed on TET1 expression (Q3/4,Q2,Q1 refer to quartile with Q1 the lowest), age, IDH1 mutation status, and histology to evaluate whether TET1 hazard is confounded by other risk factors.

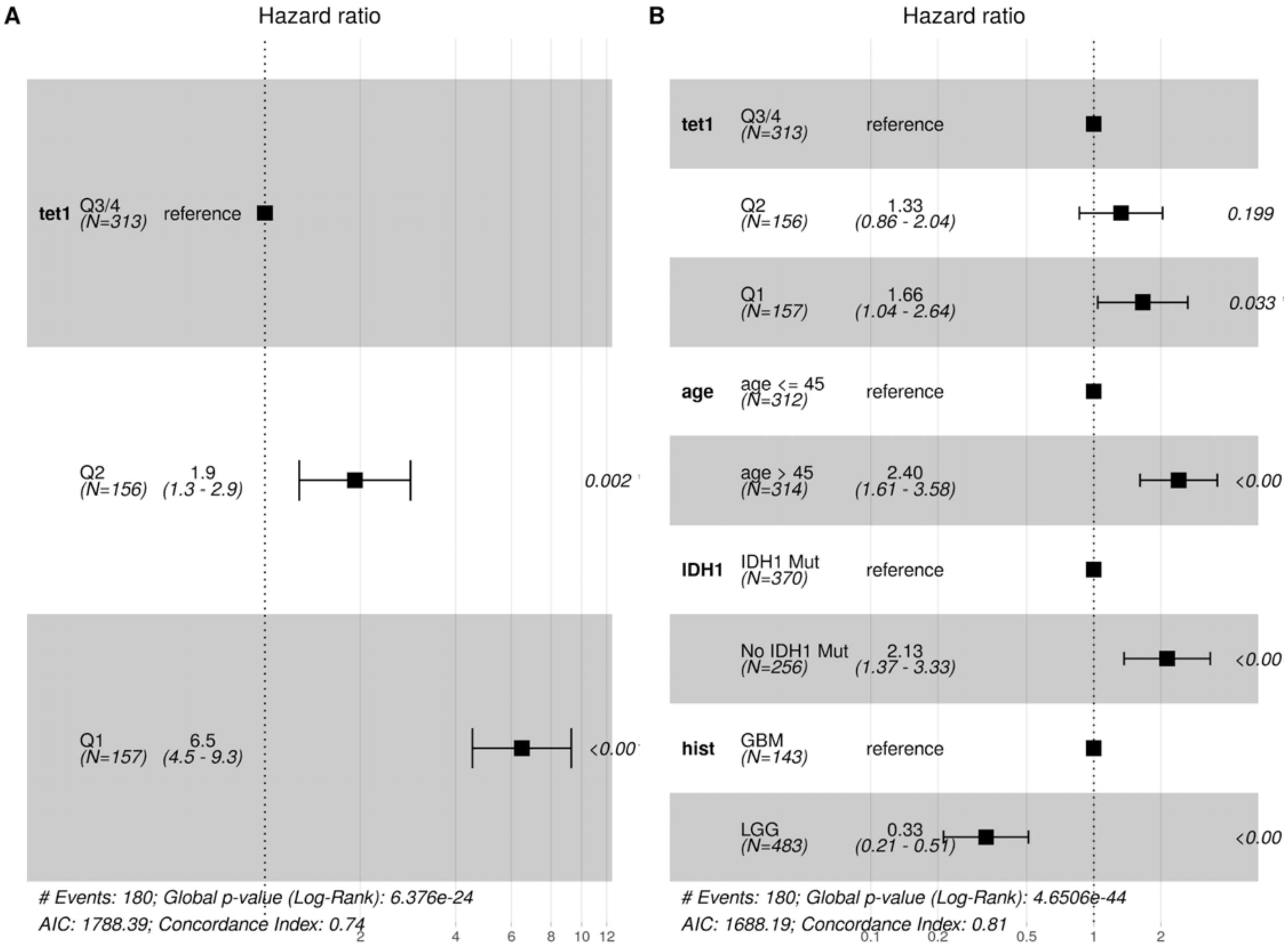

Supplemental Figure 2: (A) Cox model forest plot built on all glioma patients with TET1 quartile expression alone. **(B)** Cox model forest plot built on all glioma patients with survival as a function of TET1 quartile expression + age + IDH1 + histology.

The lowest quartile of TET1 expression is significantly associated with glioma hazard in a multivariate model of TET1 + age + IDH1 status + Histological type. The reduction in TET1 significance in the multivariate (right) relative to the univariate model (left) indicates partial dependence of TET1 survival effect on these factors.

Figure 3

Univariate Patient Set Forest Plots (Figure 1B supplement)

The below 6 forest plots track the significance and confidence intervals of TET1 hazard intervals from the 6 kaplan meier curve plots in Figure 1 of the publication. Expression quartiles are derived from the full patient set and not for each set of patients. The tet1 hazard ratio is significant at the 0.001 level for at least the lowest quartile of expression in all categories except GBM patients.

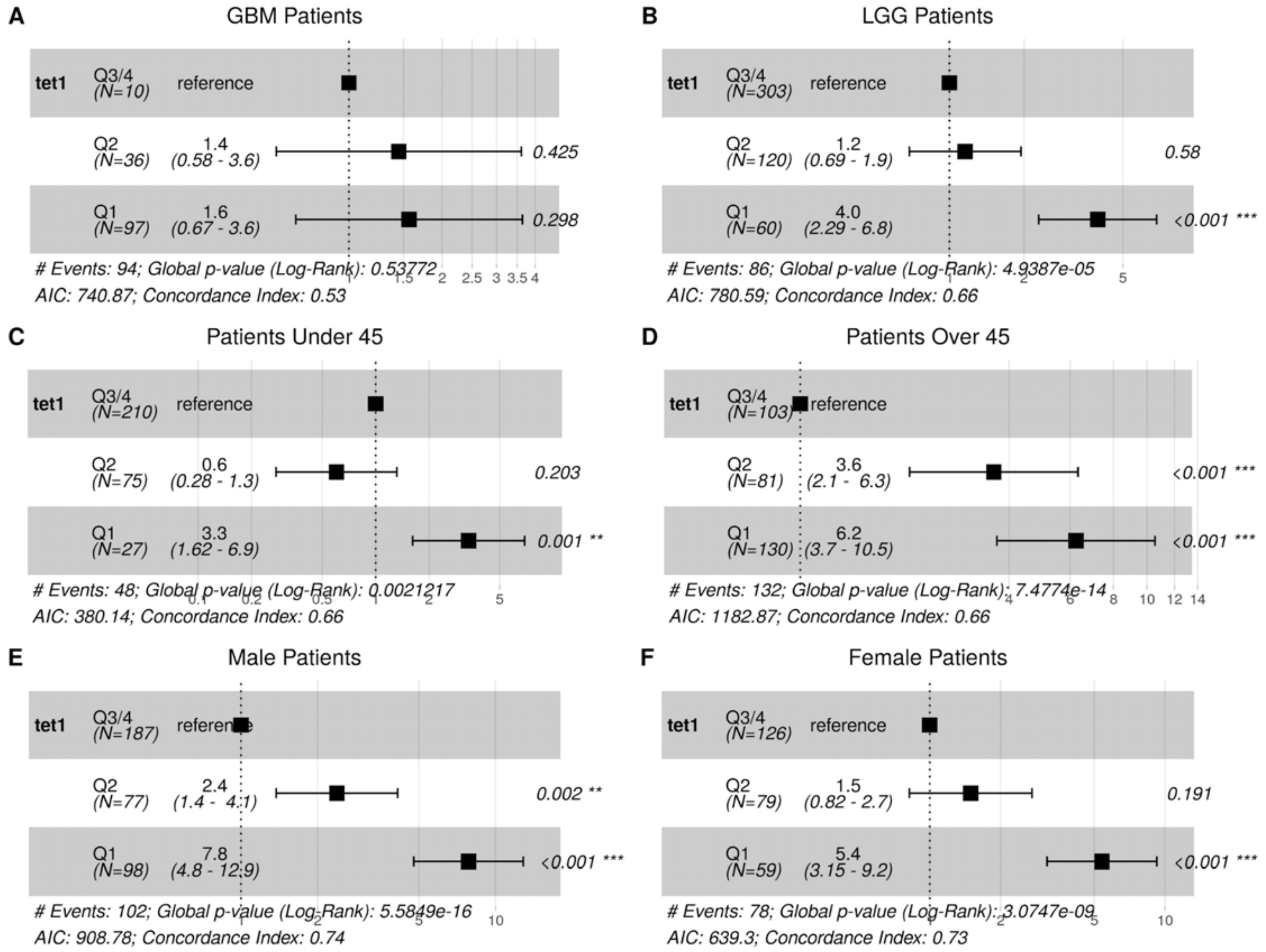

Figure 4

Multivariate Patient Set Forest Plots (Figure 1B supplement)

To further investigate whether low TET1 expression remains a risk factor after accounting for potential confounders, we reproduce the patient set specific forest plots from the last section, but use multivariate cox proportional hazards models.

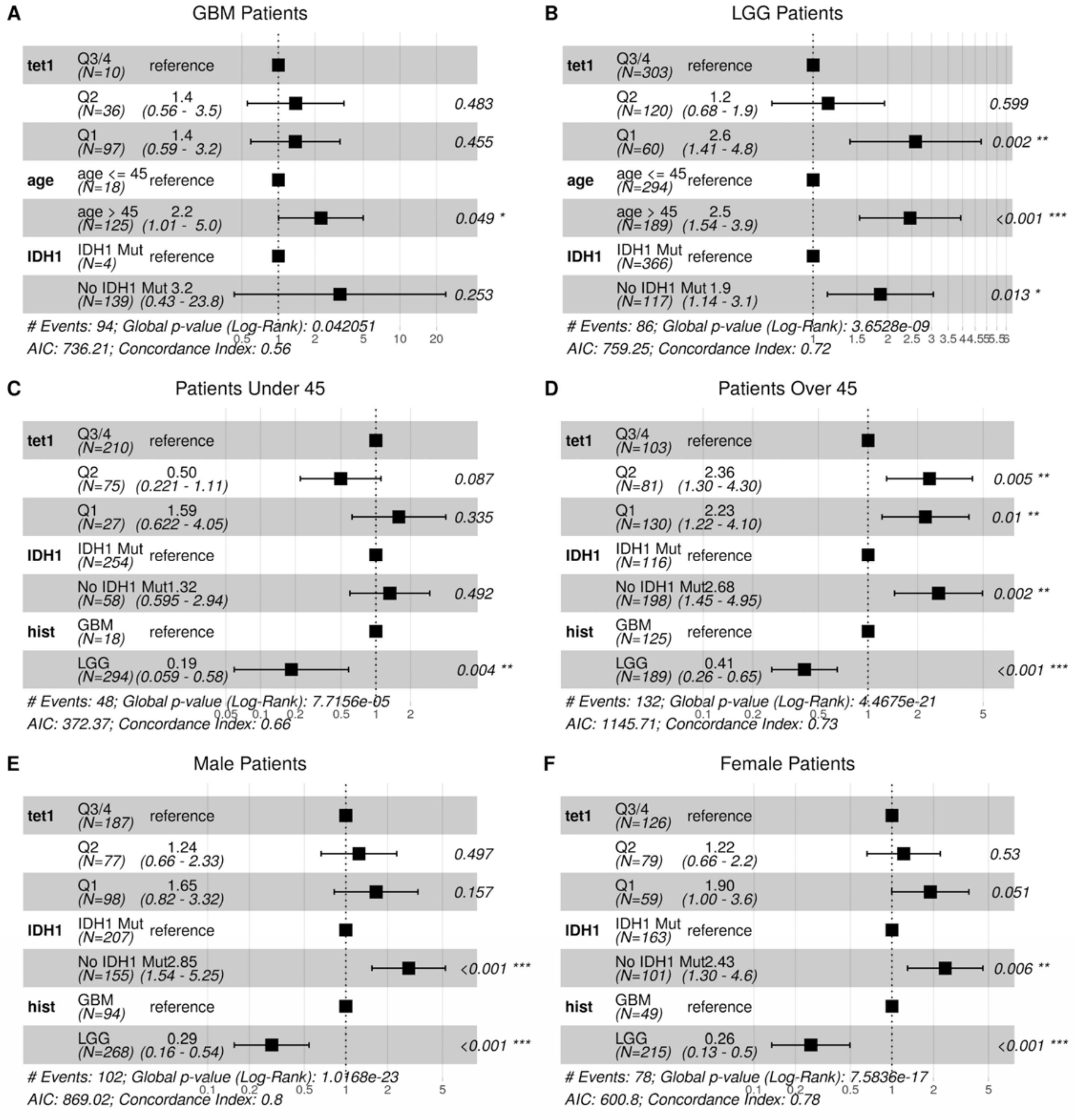

In these patient set specific multivariate cox models- low TET1 expression (Q1) remains trending towards greater hazard or significantly associated with greater hazard.

Table 2 Scratch Assay Results

Figure 4 in the publication presents a chart and image from a TET1 knockdown scratch test. Here we present some of the numeric data generated in a set of scratch test experiments.

Hours	Control (+/- S.E.M)	ShTET1 (+/- S.E.M)
4	0.76 (0.1)	0.91 (0.15)
8	0.32 (0.02)**	0.74 (0.1)*
12	0.10 (0.002)***	0.64 (0.11)*

Scratch assay numeric results from Figure 4D.

- *P < 0.05; **P <0.01; ***P < 0.001;
- Table indicates the median wound area in 3-6 experiments at each time point.
- Each value was compared to the initial cell free area
